# A data mining approach for identifying pathway-gene biomarkers for predicting clinical outcome: A case study of erlotinib and sorafenib

**DOI:** 10.1371/journal.pone.0181991

**Published:** 2017-08-08

**Authors:** David G. Covell

**Affiliations:** Information Technology Branch, Developmental Therapeutics Program, National Cancer Institute, Frederick, MD, United States of America; University of Chicago, UNITED STATES

## Abstract

A novel data mining procedure is proposed for identifying potential pathway-gene biomarkers from preclinical drug sensitivity data for predicting clinical responses to erlotinib or sorafenib. The analysis applies linear ridge regression modeling to generate a small (N~1000) set of baseline gene expressions that jointly yield quality predictions of preclinical drug sensitivity data and clinical responses. Standard clustering of the pathway-gene combinations from gene set enrichment analysis of this initial gene set, according to their shared appearance in molecular function pathways, yields a reduced (N~300) set of potential pathway-gene biomarkers. A modified method for quantifying pathway fitness is used to determine smaller numbers of over and under expressed genes that correspond with favorable and unfavorable clinical responses. Detailed literature-based evidence is provided in support of the roles of these under and over expressed genes in compound efficacy. RandomForest analysis of potential pathway-gene biomarkers finds average treatment prediction errors of 10% and 22%, respectively, for patients receiving erlotinib or sorafenib that had a favorable clinical response. Higher errors were found for both compounds when predicting an unfavorable clinical response. Collectively these results suggest complementary roles for biomarker genes and biomarker pathways when predicting clinical responses from preclinical data.

## Introduction

For over a decade claims have been made that intensive analysis of the human genome using measurements of gene expressions, mutations and single nucleotide polymorphisms (SNPs) will reveal cures for cancer. Yet as more data is generated some assert that little new biology has been revealed [1], especially when distinguishing cancer causing from bystander mutations [2], or developing therapeutic strategies based on combinations of gene signals within the overall genomic landscape[3]. Research efforts that link gene signals from preclinical studies of cultured cancer cells to outcomes from clinical trials of human cancers [4] may offer critically sought after guidance for personalized gene-directed cancer therapies [5,6].

Frequently cited strategies for linking preclinical and clinical data include a greater focus on specific ‘controlling’ components of cancer biology, such as kinase signaling or DNA repair pathways[7,8], or on developing novel informatic methods of data analysis[9,10]. Following these suggestions, the method proposed here will study agents that putatively target kinase signaling pathways, using a novel statistical analysis of publicly available preclinical and clinical data. Two data sources will be examined; i) preclinical data, derived from measures of baseline gene expressions within the Sanger Cancer Genome Project [11] (CGP, hereafter) and CGP tumor cell drug sensitivity (CGP IC_50_, hereafter) and ii) clinical data derived from pre-treatment patient baseline gene expressions and post-treatment survival data from the MD Anderson BATTLE (Biomarker-integrated Approaches of Targeted Therapy for Lung Cancer Elimination) studies[12]. The proposed goals are; i) to develop statistical models that use baseline gene expressions to link preclinical CGP IC_50_ with BATTLE clinical efficacy, ii) to extend these gene-based results to molecular function pathways and apply their associated pathway fitness scores to identify potential pathway-gene biomarkers, iii) to provide quantitative assessments of pathway-gene biomarkers as predictors of patient response, and iv) to offer literature support for the roles of model-derived pathway-gene biomarkers in compound efficacy. Although the limitations of gene expression-based methods for making successful clinical predictions have been noted[13], and, in some instances, effectively overcome by combining gene expressions with mutation status[4], the analysis proposed here will strictly adhere to using only baseline gene expressions for outcome predictions; thereby acknowledging the growing evidence that many cancers lack important genomic defects, inclusive of mutations or SNPs[2,3,14] and offering a perspective consistent with using preclinical gene expression status for personalized therapeutic strategies.

The tyrosine kinase inhibitors (TKIs), erlotinib and sorafenib, selected for the BATTLE studies, have proven survival benefits in the treatment of several cancers, including chronic myeloid leukemia, breast, liver, renal and lung cancer [15]. Erlotinib’s putative target is EGFR, while sorafenib is a multi-kinase inhibitor with reported activity against tyrosine protein kinases, such as VEGFR, PDGFR, c-Kit receptors, and serine/threonine kinases, such as C-Raf and B-Raf [16,17]. Evidence supports both compounds as multi-kinase targeting agents [18,19]. Predictive models that link erlotinib and sorafenib preclinical to clinical results (and vice versa) pose major challenges. For example, using ridge regression modeling (in the CARET package[20]), ten-fold cross-validations for predicting preclinical CGP IC_50_ from BATTLE gene expressions yielded good R^2^ values (observed versus model predicted) of 0.76 for erlotinib and 0.66 for sorafenib. Reversing this comparison found R^2^ values of 0.69 and 0.64 for erlotinib and sorafenib, respectively, for ridge regression predictions of BATTLE clinical responses, using only BATTLE gene expressions. In contrast, using preclinical IC_50_ ridge regression modeling to predict BATTLE clinical data or using clinical BATTLE ridge regression modeling to predict CGP IC_50_ yielded R^2^ values below 0.2 for each drug. These results support the need for alternative predictive models that link preclinical IC_50_ to clinical response data.

Additional challenges when linking preclinical and clinical data can be found within a recently published method [21] that reported an excellent model for predicting BATTLE patient Progression Free Survival (PFS or Months to Progression, hereafter) from preclinical CGP IC_50_. Using ridge regression modeling[22,23] based on gene expressions derived from the 15 most and 55 least sensitive CGP tumor cells, yielded an 89% classification accuracy for predicting CGP IC_50_ of the training set, a strong p-value (p<3.0e-4) separating the sensitive from insensitive tumor cells, with credible Spearman correlation statistics (rho = 0.64, p = 5.3e-4) when comparing model predictions to observed BATTLE PFS. However, small deviations in tumor cell selection in the training model, for example using a model developed from the 10^th^ percentiles of sensitive and resistant CGP tumor cells, yields poor (i.e. non-significant) model predictions of BATTLE PFS. This result suggests that while not all models trained using preclinical CGP IC_50_ data yield accurate prediction accuracies, an appropriate selection of ridge regression models based on subset sampling of existing data can yield sufficiently good predictive results to support the clinical feasibility of this approach.

Motivated by a FDA-led initiative examining poor predictions of complex endpoints, such as cancer survival using ‘curated’ gene expression biomarkers, Li *et al*. [24] proposed a novel strategy for improved model performance. Using the preclinical CGP IC_50_ and BATTLE data for erlotinib and sorafenib, their statistical prediction model applied a pathway-based gene filtering step, whereby biomarker gene selection was based on pathway linkages to these drugs’ Mechanism of Action (MOA). Using a splitting strategy between drug sensitive and resistant tumor cells, statistical training models were derived consisting of combinations of tumor cells and gene expressions that ‘capture consistent biomarker features across their training dataset’ (a panel of 240 human cancer cell lines, www.Eurofinspanlabs.com). Validation of their approach found clinical prediction accuracies comparable to Geeleher *et al*.[21]. Although the steps for pathway-based gene filtering and tumor cell selection were not precisely provided, the benefit of pathway-based gene selection for subset sampling of the complete dataset appears to be advantageous for model prediction.

Building from these previous results, a data mining strategy is proposed that develops robust preclinical training models for clinical prediction. This strategy uses large-scale random sampling of i) training models that strongly correlate preclinical CGP IC_50_ predictions with model-averaged gene expressions, and ii) test models, using patient gene expressions applied to each training model, that also yield clinical predictions that strongly correlate with BATTLE clinical outcome. Biomarker genes are selected from models satisfying these joint criteria and on their appearance in GO:molecular function pathways. A qualitative assessment of treatment prediction accuracies for these biomarker genes is provided. The results will include;

1. identification of a subset of potential biomarker genes using correlative measures of goodness of fit for linear ridge model predictions of preclinical CGP IC_50_ and BATTLE clinical outcome
2. analysis of linear ridge derived biomarker genes using
  - traditional statistical methods based on comparing genes within the distribution tails (i.e. sensitive and resistant tumor cells) for preclinical CGP IC_50_ and BATTLE patients with the best and worst clinical outcome
  - pathway-gene clustering of results from Gene Set Enrichment Analysis (GSEA)
  - application of pathway fitness scores for identifying important targeted pathways and their genes (e.g. pathway-gene biomarkers)
3. development and quantitative assessment of predictors for BATTLE clinical outcome from pathway-gene biomarkers based on
  - Random Forest(RF)-derived prediction errors
  - Receiver-Operator-Character (ROC) analysis

Collectively, these results will be shown to yield reliable predictive models of BATTLE clinical outcome using preclinical CGP IC_50_ data.

## Methods

### Data availability

The CGP data is publically available from http://www.cancerrxgene.org/downloads. The BATTLE microarray and patient response data are publically available as Series GSE33072 in the Gene Expression Omnibus (https://www.ncbi.nlm.nih.gov/geo/)).

### Linear ridge regression

Linear ridge regressions were completed using the CRAN R package (ridge::linearRidge), applying the ridge parameter selection method of Cule and De Lorio[25], on unscaled data. CGP IC_50_ values were available in 258 and 285 tumor cells for erlotinib and sorafenib, respectively; with 11,582 and 11,884 gene expressions from these tumor cells mutually available in the CGP and BATTLE datasets. As a reference, linear ridge modeling using the complete set of CGP IC_50_ and CGP gene expressions yielded Pearson correlation coefficients of model IC_50_ predictions versus CGP IC_50_ values of 0.91 (p = 2.2e-16) for erlotinib and 0.65 (p = 2.1e-16) for sorafenib. In contrast, predictions of BATTLE clinical results using CGP IC_50_ derived linear ridge models were quite poor, yielding Pearson correlation coefficients of -0.27(p = 0.19) and 0.16 (p = 0.33) for erlotinib and sorafenib, respectively. These results are consistent with the previously discussed CARET-based finding of poor clinical predictions using preclinical CGP IC_50_ data. In addition, Pearson correlations of CGP IC_50_ values with each tumor cell’s gene expressions finds 1477 genes for erlotinib (p < = 0.05, FDR corrected) and only 1 gene for sorafenib. Although the former number of genes is manageable for pathway analysis, the latter is not.

Following the designs of Geeleher et al. [21] and Li et al.[24] (and their apparent success), subsets of CGP tumor cells and their gene expressions were analyzed. Simulations (N = 20 X 10^6^), arbitrarily based on 20, 30 and 50 tumor cells and 200, 300 and 500 gene expressions, were completed. Due to the large numbers of regression samples and the relatively shorter compute times of linear versus logistic regression; the former was chosen for this analysis. The goal is to develop predictive models of preclinical CGP IC_50_, using CGP gene expressions, then apply this model, using the clinical gene expressions of these same genes, to predict BATTLE clinical responses. Model evaluations are based on statistical p-values from correlative fittings of model-derived predictions of preclinical CGP IC_50_ data and BATTLE clinical patient response data. Two levels of correlative comparisons are made. The first uses the p-value for the Pearson correlation of each linear ridge regression training model’s predicted preclinical CGP IC_50_ against the model’s gene expressions, averaged across each tumor cell in the training model (referred to hereafter as log(pval_IC_50_)). The second correlative comparison is based on the p-value of Pearson correlations between the test model’s prediction of BATTLE clinical response, using BATTLE patient-derived gene expressions in the training model, and the observed clinical response, (referred to hereafter as log(pval_clinical)). Correlative comparisons required the range of model predicted CGP IC_50_ and model predicted BATTLE patient responses to be at least 80% of their observed values.

### Gene Set Enrichment Analysis

Gene Set Enrichment Analysis (GSEA [26]) will be used to identify pathways associated with subsets of genes identified from linear ridge regression analysis. GSEA results are limited to only pathways with at least 2 shared genes; with application of a False Discovery Rate (FDR) against a chance finding at the typical threshold of 0.05. GSEA reporting will be restricted to the topmost significant (FDR q-score) pathways and will emphasize recurrent biological themes for these pathways rather than individual pathways. GSEA will be restricted to only the GO:molecular function ontology, which involves task-related genes that function in transport, binding and modifying molecules (e.g. phosphorylation) within the cell. These tasks are regarded here as appropriate for examinations of pathways relevant to these therapeutic compounds. See http://geneontology.org/page/molecular-function-ontology-guidelines for a complete description of the GO:molecular function ontology.

### Pathway fitness scores

Pathway fitness scores (H) are based on modifications to a previously developed method described in Huang *et al*.[27]). This calculation is based on the t-statistic testing the significance of differential tumor cell gene expressions between the upper and lower percentiles of Months to Progression for BATTLE patients (referred to hereafter as the responder and non-responder patients, respectively). The t-statistic for genes in a pathway and genes not in a pathway are compared as two sample populations using the Kruskal–Wallis rank sum procedure. H is generated using the rank sum for all correlation coefficients, then assessing the ranks for correlation coefficients of genes in the pathway versus genes not in the pathway. Pathway fitness represents a quantitative measure of concordance for within pathway gene expressions when compared to all non-pathway gene expressions (see **Appendix,** Huang *et al*. [27] for further details). A large absolute value of H indicates a strong difference between the two sample populations. A positive H indicates pathway gene expressions that are mostly over expressed in the responder versus non-responder BATTLE patients. A negative H indicates pathway gene expressions that are mostly over expressed in the non-responder versus responder BATTLE patients. Important genes can be assessed according to their contribution to the total pathway fitness score by recalculating H in the absence of each pathway gene (i.e. leave-one-out). The relative contribution of each gene to H, referred as delta(fitness), represents the averaged contribution to pathway fitness scores for all pathways having this gene. Ordering these results according to delta(fitness), then selecting the extreme (positive and negative) values provides a means to identify only the topmost genes contributing to H. Reported values of delta(fitness) will be limited to pathway gene expressions showing a modest (p< = 0.2) correlation with clinical outcome.

### Random Forests

Random Forests (RF) will be used to rate how well gene expressions, selected jointly from correlative statistics and pathway fitness scores, predict patient response. Introduced in 2001 by Leo Breiman[28,29], RF function as an ensemble learning method based on the aggregation of many decision trees. The general idea is to build a large number of decision trees using a subset of random samples from the training data (referred to as bagging for *bootstrap aggregation*) then use a simple majority-rule vote for final decision making. The concept of aggregating the results of many decision trees has resulted in a stable algorithm, robust to noisy data[29]. A useful analogy of this process would be an orchestra composed of 1^st^, 2^nd^ and 3^rd^ musicians in the brass, woodwind, and percussion sections. Any one musician’s mistake is less apparent, since what you hear (i.e. your decision about the music) is based on many inputs. RF split the data into the *sample*, *validation* and *test* datasets. Default RF build 500 trees using a randomly *sampled* subset of the data. The *validation* dataset has not been used to build the specific model but to see whether the model is better or worse than the previous model. Once satisfied with tuning in terms of the *validation* dataset the model is applied to the *test* dataset for the final unbiased estimate of prediction error. RF calculations are implemented in the R-package, using the RATTLE utility http://rattle.togaware.com/ for defining RF parameters and sampling, validation and testing steps.

Two issues are important when assessing RF predictions. The first is due to RF inherent random sampling of the data; which produces different results for each RF calculation (unless the same seed is selected). This issue can be addressed by averaging RF predictions across many simulations. The second issue involves class assignment; RF error rates for correct prediction requires *a priori* class assignment, here, to either a responder or non-responder class. Class assignments were determined by optimizing RF prediction errors for different splits of the response data into responder and non-responder groups. These results define the optimal boundary of class assignment for assessing the role of sample size in prediction errors.

### Cytoscape analysis

To provide an alternative perspective of the results obtained here, pathway fitness scores and pathway genes will be visualized by generating a Cytoscape [30] network interaction map. The steps to accomplish this are;

1. build a non-redundant pairwise set of pathway genes selected from gene sets derived from GSEA pathways
2. calculate all pathway fitness scores
3. select the upper and lower 20^th^ percentiles pathway fitness scores
4. weight pairwise pathway genes with their pathway fitness score
  - generate a force directed network model using these weights

## Results

### Random sampling—Linear ridge modeling

**Fig 1:**
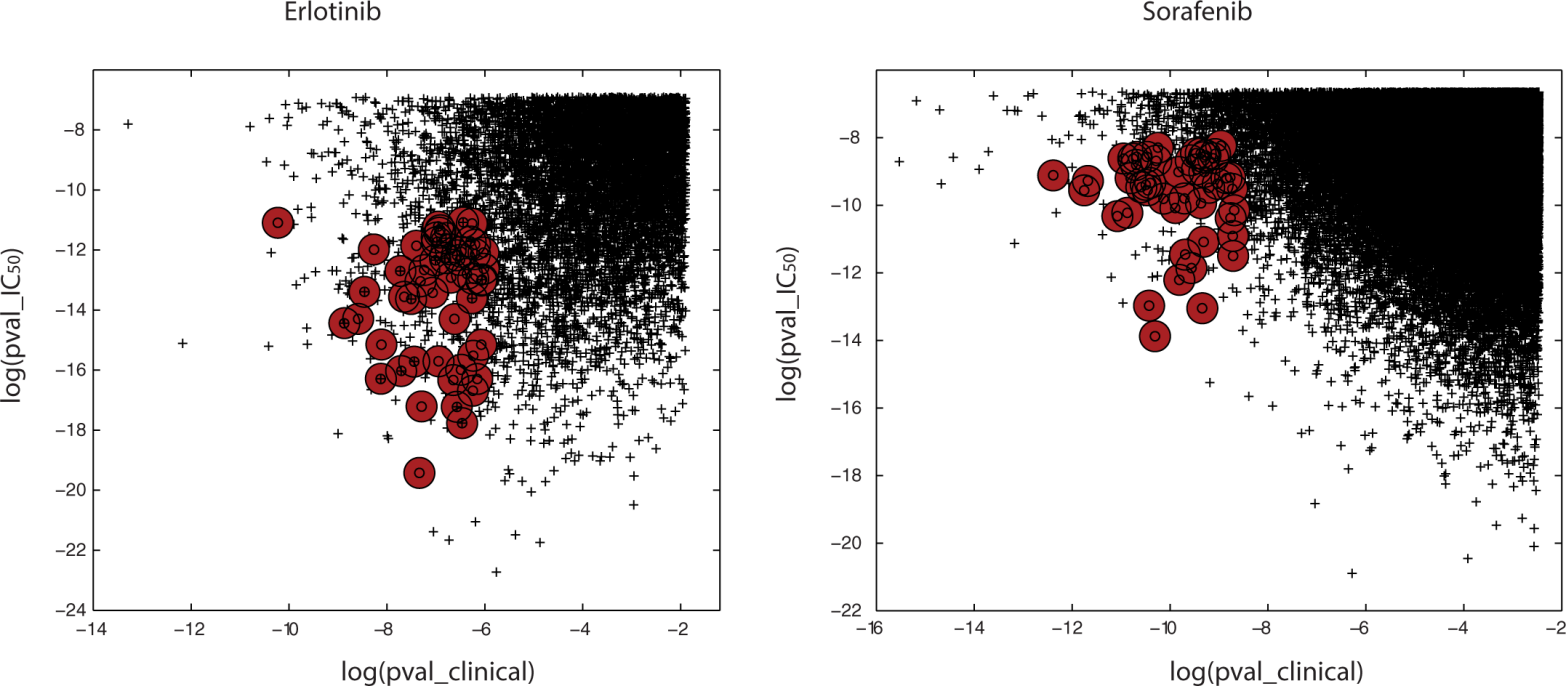
Erlotinib (left panel) and sorafenib (right panel) for log(pval_clinical) of the Pearson correlation coefficient for each training model’s prediction of the clinical response(x-axis) versus the log(pval_IC_50_) for the correlation coefficient of each model’s prediction of IC_50_ versus the mean of each gene’s expression in the training model (y-axis). These results represent 20 million random picks of 30 tumor cells and 300 genes from the CGP database of IC_50_ values for erlotinib and sorafenib. For erlotinib, only 53 simulations achieved the arbitrary threshold requirements of log(pval_IC_50_) < -11, log(pval_clinical) < -6, ppv_clinical_ < 0.45 and npv_clinical_<0.45 and. These models appear as the red circles in the left panel. For sorafenib only 48 simulations achieved the threshold requirements of log(pval_IC_50_) < -8.5, log(pval_clinical) < -8.5, ppv_clinical_ < 0.65 and npv_clinical_ < 0.65). Ppv and npv calculations require selection of a boundary between good and poor responses. These calculations use the mean of the predictive values as this boundary. Evident from this figure is the occurrence of training models with excellent correlative statistics that fail to meet the thresholds for ppv and npv.

The results plotted in **Fig 1**display, on the x-axis, the log(pval_clinical) for the correlations of model predicted versus clinical outcome and, on the y-axis, the log(pval_IC_50_) for the correlation of model predicted preclinical CGP IC_50_ versus tumor averaged gene expressions. These results represent 20 million simulations based on a training model using random samplings of 30 tumor cells and 300 genes. For reference there are n!/r!(n-r)! ~ 10^41^ combinations for selecting cells and ~10^600^ combinations for selecting genes. Admittedly, compromising with 20 million samples represents a very small coverage of the complete cell-gene space. Notable in **Fig 1**is the presence of training models that have good log(pval_IC_50_) for preclinical CGP IC_50_ prediction and poor log(pval_clinical) for clinical prediction, and vice-versa. Extrapolating these results to Geeleher *et al*. [21] and Li *et al*. [24] supports the existence of good training models of preclinical CGP IC_50_ producing broadly variable results for goodness of fit to BATTLE clinical outcome. Cross validation of model predictions based on training and test subsets derived from each of the 30–300 cell-gene samplings were not completed in this analysis. Cross-validated models may shorten calculation times by eliminating the need for assessing pval_IC_50_ and pval_clinical, however are not expected to significantly influence the reported results.

The adaptation proposed here, to improve the limitations related to variations in prediction accuracy, incorporates the quality of clinical prediction when selecting the most appropriate samplings of gene-cell combinations for linear ridge regression. Rather than selecting one training model then assessing its performance for clinical prediction and reporting only the ‘best’ results, a random selection of gene-cell combinations is used to build each training model, which in turn is tested for goodness of clinical prediction. This strategy is supported by inspection of **Fig 1**, where relatively few instances exist for good log(pval_IC_50_) and good log(pval_clinical) (shown as red circles in **Fig 1**). A*d hoc* thresholds were adjusted for log(p-values) that define goodness of model correlative fits, combined with inclusion of positive and negative predictive power (ppv_clinical_ and npv_clinical_, respectively) for clinical response, to yield a relatively small number of training models (53 training models for erlotinib and 48 training models for sorafenib; see **Fig 1**caption for details). This adaptation serves to eliminate false positive training models (i.e. training models that have excellent correlations of predicted preclinical CGP IC_50_ to model-averaged gene expressions, yet yield poor predictions of clinical outcome, and vice-versa). Hereafter, this joint strategy for model selection will be referred to as dual filtering.

Results for linear ridge regression models using alternative sizes of gene-cell combinations found that smaller numbers of genes (n = 200) and tumor cells (n = 20) yielded results qualitatively similar to those displayed in **Fig 1**, yet with surprisingly few hits sharing a low log(pval_IC_50_) and a low log(pval_clinical). Models based on larger numbers of gene(n = 500)-cell(n = 50) combinations, with 20 million simulations and the same threshold for model acceptance as used in **Fig 1,** yielded no hits comparable (to the 30–300 cell-gene model) for either erlotinib or sorafenib. This result may be due in part to the considerably larger gene-cell space, when compared to using 300 genes and 30 cells, and the need for greater than 20 million randomly-chosen samples. In summary, alternative numbers of gene-cell combinations for linear ridge models may yield slightly different results, however it is believed that using 300 genes and 30 tumor cells represents a reasonable compromise for adequately sampling gene-cell space that yield numbers of tumor cells and genes comparable to Geeleher *et al*. [21] and Li *et al*.[24]. Extensive simulations (>20 million) using randomized data produced no hits with log(p-values) below the dual filtering thresholds described in the caption to **Fig 1**. This result is not surprising since it is unlikely that a training model based on randomized CGP IC_50_ values would yield significant correlative statistics. The importance of this result supports the claim that models jointly sharing strong values for pval_IC_50_ and pval_clinical are distinct from randomly selected cell-gene combinations.

### Erlotinib—Linear ridge modeling

**Fig 2:**
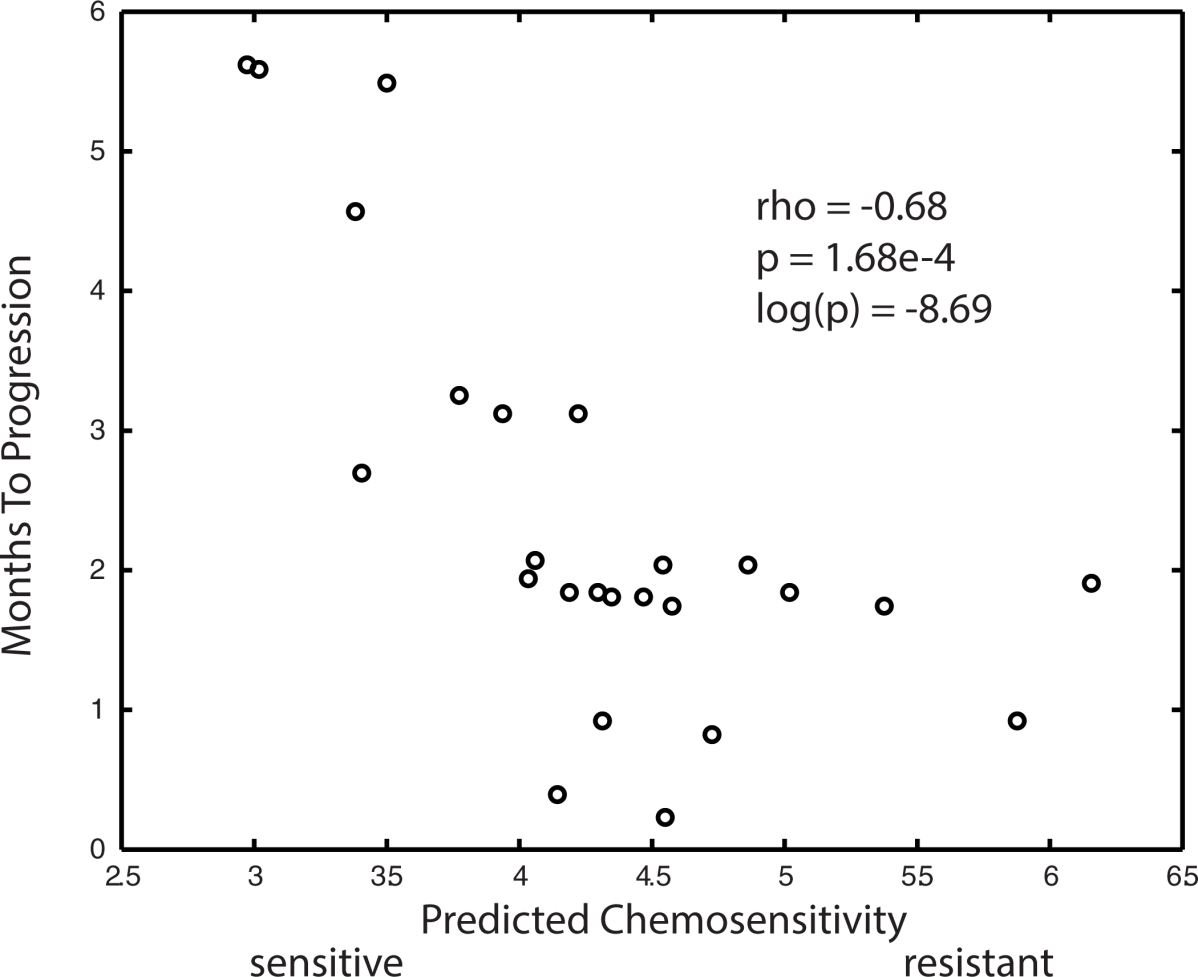
Plot represents the average performance of the 53 linear ridge models selected by dual filtering of random simulations based on goodness of fit of the predicted preclinical erlotinib IC_50_ data with model averaged gene expressions (training data), and goodness of fit to the clinical outcome of BATTLE patients receiving erlotinib (test data). X-axis represents the model predicted chemosensitivity when using the BATTLE gene expressions in the training model. Y-axis represents the 25 patient’s observed Months to Progression. Correlative statistics appear in the upper right.

**Fig 2**plots the Months to Progression for the 25 patients in the BATTLE study versus the average predicted chemosensitivity for the 53 training model’s predictions of the test data. The Pearson correlation for this plot is -0.68, with a p-value for significance of 1.68e-4. On average, this correlation achieves a statistical significance exceeding that found by Geeleher *et al*. [21] and Li *et al*.[24]. The performance statistics for the 53 accepted regression training models are listed in **S1 Table**. In summary, an average correlation coefficient of -0.62 was observed for each training model’s prediction versus observed Months to Progression for the BATTLE data, with an average log(p-value) of -6.97 (p = 1.20e-3). Average ppv_clinical_ and npv_clinical_ values for these models were 0.54 and 0.79, respectively. Pearson correlations of model predicted CGP IC_50_ to observed CGP IC_50_ were all above 0.97, with p-values in the 10^−14^ to 10^−19^ range. An average correlation coefficient of 0.75 was observed for the training model’s prediction of CGP IC_50_ versus the model-averaged gene expressions, with an average log(p-value) of -13.58 (p = 4.00e-6).

Fisher’s exact statistics were used to assess whether the 53 linear ridge models were enriched for tumor cell type (n = 258) or tumor tissue type (n = 17). Sixty-two percent (161/258) of the tumor cells screened against erlotinib were included in at least one in the 53 linear ridge models. A Fisher’s exact test finds the most statistically significant tumor cell enrichment for TE-12 (upper_aerodigestive) (p<0.0037), OS-RC-2 (renal) (p<0.0122), TE-6 (upper aerodigestive) (p<0.0122), TK10 (renal) (p<0.012), LB996 (renal) (p<0.0298), EW-12 (bone) (p<0.033) and NCI-H2171 (lung) (p<0.033). Assessing enriched tumor tissue types finds blood (p<0.00147) and kidney (p<0.000833) to be enriched. For reference, Fisher’s exact tests for tissue type enrichment within the 50 most erlotinib sensitive tumor cells finds enrichment for only renal (p<0.048) tissue, while only lung (p<0.0046) and blood (p<0.015) were enriched in the 50 most erlotinib resistant tumors. These results support a slight enrichment in renal tumors for the linear ridge models with a broad sampling of all tumor types.

### Erlotinib—Statistical analysis for potential biomarker genes

Over 3k genes appear in the topmost 53 training models, with 741 genes existing in at least two of these training models. These 741 gene expressions constitute potential biomarker genes for jointly separating erlotinib CGP IC_50_ chemo-sensitive from chemo-resistant responses, and erlotinib treated BATTLE responders from non-responders. These 741 genes can be analyzed using traditional Student’s t-tests, based on comparisons of the upper and lower 30^th^ percentiles of preclinical CGP IC_50_ and BATTLE patient responses. Two-hundred and twenty-four differential gene expressions (of the 741 regression-derived genes) are found based on a Student’s t-test (p<0.05) using erlotinib’s CGP IC_50_’s sensitive versus resistant responses and 129 differential gene expressions are found based on Student’s t-tests comparing erlotinib’s BATTLE patient responders to non-responders. However, these gene sets fail to intersect. In summary, GSEA pathways are found that associate preclinical CGP IC_50_ chemo-resistance and poor BATTLE patient responses to TRANSPORTER pathways, and preclinical CGP IC_50_ chemo-sensitivity and better BATTLE patient responses to GSEA pathways involving known targets of erlotinib; including LIGASE, OXIDOREDUCTASE and DIMERIZATION associated pathways. Within this gene set is EGFR, which functions in ubiquitin protein ligase binding and protein dimerization, and is also consistent with erlotinib targeting the oxidoreductase activity of cytochrome P450 (http://www.drugbank.ca/drugs/DB00530). Although these results fail to identify a common set of genes as potential biomarkers, GSEA identifies common GO:molecular function pathways that associate erlotinib preclinical CGP IC_50_ with BATTLE clinical outcomes. A detailed discussion of this analysis appears in **S1 Text.**

### Sorafenib- Linear ridge modeling

**Fig 3:**
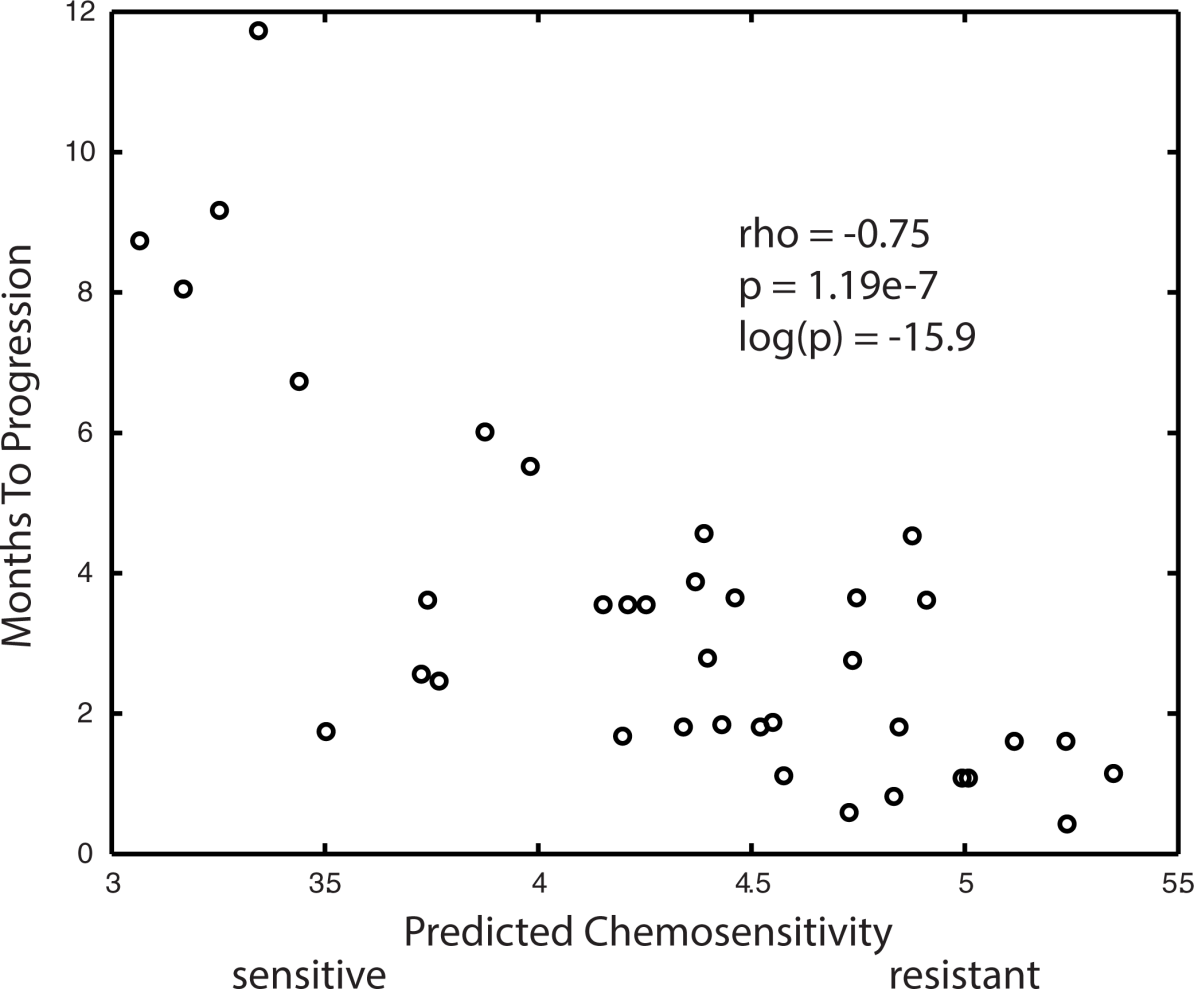
Plot represents the average performance of the 48 linear ridge models selected by dual filtering of the random simulations based on goodness of fit of the predicted preclinical sorafenib IC_50_ data with averaged gene expressions (training data) and goodness of model fit to the clinical outcome for 37 BATTLE patients receiving sorafenib (test data). X-axis represents the model predicted chemosensitivity when using the BATTLE gene expressions in the training model. Y-axis represents the patient’s observed Months to Progression. Correlative statistics appear in the upper right region of the plot.

**Fig 3**plots the Months to Progression for the 37 BATTLE patients receiving sorafenib versus the average model predictions of chemosensitivity for the 48 linear ridge models passing the p-value thresholds for goodness of fit to each dataset. The Pearson correlation coefficient for the model predictions versus Months to Progression of -0.75 is highly significant (p = 1.19e-7). The statistics for the performance of the 48 accepted training models are listed in **S2 Table**. In summary, an average Pearson correlation coefficient of -0.61 was observed for the linear ridge model’s prediction of Months to Progression to that observed from the BATTLE clinical data. The average p-value for these correlations was 8.26e-5 (log(p-value) = -9.71), with averages of 0.69 and 0.74 for ppv_clinical_ and npv_clinical_, respectively. The Pearson correlations of model prediction to observed CGP IC_50_ values were all above 0.97, with p-values in the 10^−14^ to 10^−19^ range. An average correlation coefficient of 0.67 was observed for the training model’s prediction of CGP IC_50_ and the model’s averaged gene expression values, with an average log(p-value) of -9.98 (p = 7.82e-5). Fisher’s exact statistics were used to assess whether the 48 linear ridge models were enriched for tumor cell type (n = 285) or tumor tissue type (n = 17). Seventy-eight percent (223/285) of the tumor cells screened against sorafenib were included in at least one in the 48 linear ridge models. Fisher’s exact tests for tumor cell enrichment of these linear ridge models finds enrichment for HOP-62 (NSCLC:adenocarcinoma) (p<0.0049), D-247MG (glioma) (p<0.0071), MRK-nu-1 (breast) (p<0.0098), OS-RC-2 (renal) (p<0.0135), TE-5 (upper_aerodigestive) (p<0.0159), J-RT3-T3-5 (leukemia) (p<0.0186) and SR (blood) (p<0.0186). Assessing enriched tumor tissue types, however, finds only lung (p<0.032) to be enriched. For reference, Fisher’s exact tests for tissue type enrichment within the 50 most sorafenib sensitive tumor cells finds blood (p<0.0018) and lung (p<0.0315) tissues to be enriched, while only lung tumors (p<0.013) were enriched in the 50 most sorafenib resistant tumors. These results support a slight enrichment in lung tumors for the linear ridge models and a broad sampling of all tumor types.

### Sorafenib—Statistical analysis for potential biomarker genes

As above with erlotinib, the aim is to identify subsets of gene expressions that separate sorafenib chemo-sensitive from chemo-resistant preclinical CGP IC_50_, and also separate sorafenib treated BATTLE responders from non-responders. Eight-hundred and fifty-one genes represent the most frequently occurring genes in sorafenib’s 48 linear ridge models. Comparing the upper and lower 30^th^ percentile of significance scores finds 104 differential gene expressions (of the 851 genes) based on sorafenib’s preclinical CGP IC_50_’s and 90 differential gene expressions based on sorafenib’s BATTLE patient responses, with only 11 genes in common. Summarizing these results; genes relatively over expressed in the chemo-sensitive versus chemo-resistant CGP IC_50_ tumor cells finds GO:molecular function pathways for RECEPTOR, TRANSFERASE and LIGASE ACTIVITY. No GSEA overlaps were found for differentially expressed genes associated with CGP IC_50_ chemo-resistance. In contrast, GSEA for genes relatively over expressed in BATTLE responders versus non-responders finds GO:molecular function pathways; RECEPTOR ACTIVITY and LIGAND_DEPENDENT_NUCLEAR_RECEPTOR_ACTIVITY. Genes relatively over expressed in BATTLE non-responders versus responders are associated with DNA BINDING and KINASE BINDING pathways. A broad interpretation of these findings suggests an association between sorafenib BATTLE responders and CGP IC_50_ chemo-sensitivity; for pathways associated with over expressed genes known to be targets of sorafenib. As observed with erlotinib, however, this type of analysis yields useful results, yet fails to find a unified set of biomarker genes that establish linkages between preclinical CGP IC_50_ data and BATTLE clinical responses. A detailed discussion of this analysis appears in **S2 Text.**

### Erlotinib—GSEA for 741 genes derived from linear ridge regression

The 741 genes derived from linear ridge regressions for erlotinib are common to only a small fraction of genes derived when applying the traditional Student’s t-tests described above, yet, these genes are jointly associated with strong correlations of preclinical CGP IC_50_ to model-averaged gene expressions and strong correlations of model predicted to BATTLE observed clinical responses (cf. **Fig 1**). GSEA of these 741 genes finds two broad categories: one consisting of TRANSPORTER_ACTIVITY and the other consisting of KINASE_ACTIVITY. Given that erlotinib is a TKI (tyrosine kinase inhibitor) it is reassuring to find TRANSMEMBRANE_RECEPTOR_PROTEIN_TYROSINE_KINASE_ACTIVITY within the GO:molecular function pathways having the lowest FDR q-values for this gene set (see **S3 Table** for the complete list of GSEA pathways). Furthermore, seven additional GSEA pathways are found that represent MEMBRANE_KINASE_ACTIVITY; inclusive of pathways involved in TRANSFERRING_PHOSPHORUS_CONTAINING_GROUPS. The other category of GO:molecular function pathways, consisting of TRANSPORTER_ACTIVITY, appears in six GSEA entries.

**Fig 4:**
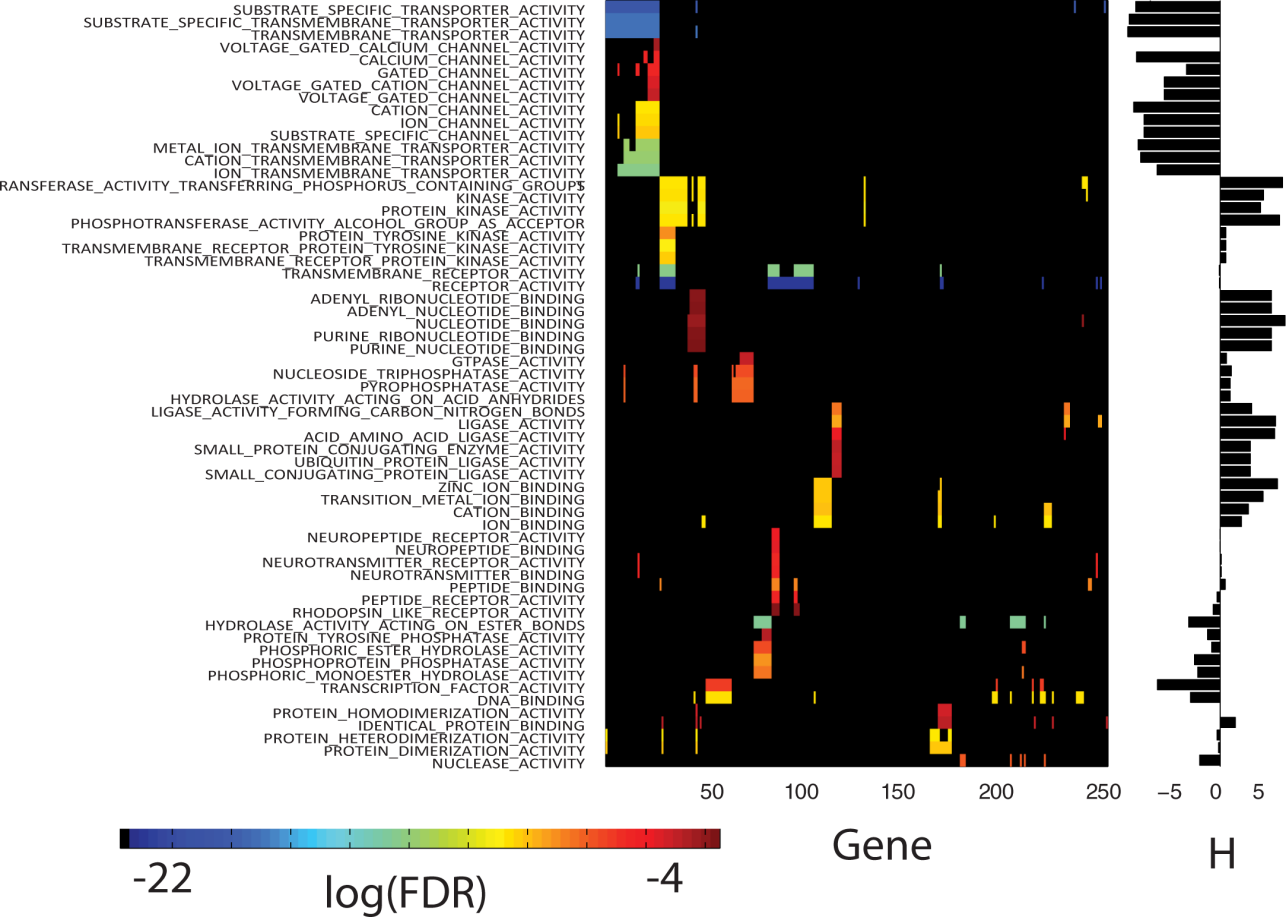
Clustered plot of the topmost significant GSEA pathways and the genes appearing in each pathway. Rows (GSEA Pathways) and columns (one’s for genes in each pathway, zeroes otherwise) have been clustered using a Minkowski’s distance metric and Wards linkage. Pathway genes in each row are colored spectrally by their log(FDR q-value), (blue to red, most to least negative) as listed in **S3 Table**. FDR q-values were not used for clustering, only the presence or absence of pathway genes. The top 61 GSEA pathways were arbitrarily selected for display to enhance readability of labels. The complete clustered plot for all significant (FDR <0.05) GSEA pathways appears in **S2 Fig**. The pathway fitness scores, H, appear in the vertical bar plot at the right. A minimum of 5 pathway genes are required for a non-zero fitness score.

Two-hundred and forty-one of the 741 genes exist within topmost 100 GSEA pathways having a FDR q-value below 0.05. These pathway-gene associations can be clustered (Minkowski distance metric, Wards linkage) in both dimensions; where pathway genes are assigned a one for present and zero otherwise. Clustering consolidates GSEA pathways having the most similar gene members and genes having the most shared appearance in pathways. **Fig 4**displays the clustered results for the subset of pathway-gene clusters having the highest overlap of shared members (see **S1 Fig** for the clustered plot of all 100 pathway-gene associations). Pathway-gene clusters (referred to hereafter as meta-clusters) are organized from top to bottom in **Fig 4**. The first meta-cluster consists of TRANSPORTER and CHANNEL_ACTIVITY related GO:molecular functions (rows 1–14). The second meta-cluster consists of KINASE_ACTIVITY, NUCLEOTIDE_BINDING, GTPase, PHOSPHATASE and HYDROLASE pathways (rows 15–29). Each of these meta-clusters share common genes, however, few genes are common to both meta-clusters. GSEA pathways with poorer FDR q-values, found in the lower portion of **Fig 4**, involve less similar sets of pathway genes when compared to the groups near the top. However, the LIGASE (rows 34–38), HYDROLASE (rows 50, 52 and 54), TRANSCRIPTION and DNA BINDING (rows 55,56) and DIMERIZATION (rows 59,60) pathways may also represent molecular functions important for the efficacy of erlotinib. The erlotinib biomarker genes EGFR and ALK [31] appear in pathways found in rows 15–29, with EGFR also found in the DIMERIZATION pathways. Overall, 6 tyrosine kinases (EGFR, KDR, LTK, ALK, ROR1 and TIE) appear in pathways found in rows 15–23. Although noted as a tumor suppressor when mutated[32], ARID1A’s appearance in the DNA BINDING pathway is consistent with the potential role of chromatin remodeling in selected cancers[33,34].

Pathway fitness scores are shown as the vertical bar plot at the right of **Fig 4.** These results find negative H values to be associated with meta-cluster(rows 1–14) (TRANSPORTER and CHANNEL_ACTIVITY) and meta-cluster(rows 48–56) (HYDROLASE, PHOSPHATASE, TRANSCRIPTION and DNA BINDING ACTIVITY) and positive H values to be associated with pathways for KINASE_ACTIVITY, meta-cluster(rows 15–23), NUCLEOTIDE_BINDING, meta-cluster(rows 24–28), PHOSPHATASE and GTPase_ACTIVITY, meta-cluster(rows 29–32), LIGASE_ACTIVITY, meta-cluster(rows 33–38) and ION_BINDING, meta-cluster(rows 39–42).

**Table 1**summarizes genes identified as contributing the most to each pathway fitness score. An illustration of using positive and negative pathway fitness scores for identifying potentially important genes is provided here. Using as an example the meta-cluster (rows 15–23) with positive pathway fitness scores. All 23 genes in this meta-cluster are in the TRANSFERASE_ACTIVITY_TRANSFERRING_PHOSPHOROUS_CONTAINING_GROUPS pathway, with 40% or more of these genes appearing in the other 8 pathways in this meta-cluster. The genes associated with the 9 pathways in this meta-cluster finds STK11, STK10, MPP3, LTK, DGKE, HIPK3, MARK1 and CPNE3 as contributing the most to pathway fitness scores. **Fig 5**summarizes these results. The bottom 4 genes in this list are relatively under expressed in the responder versus non-responder patients, while the top 4 genes are relatively over expressed in the responder versus non-responder patients. Literature supports roles for these genes in erlotinib efficacy. **STK11**(also known as LKB1)-deficient cells exhibit enhanced sensitivity to erlotinib *in vitro* and *in vivo*, an effect associated with alterations in energy metabolism and mitochondrial dysfunction, resulting in impaired ATP homeostasis and increased ROS [35]. Relative **STK11** under expression in responders versus non-responders is consistent with this finding. **LTK** shares a high degree of homology (nearly 80% identical) with ALK [51,52] and is thought to promote growth and survival through activation of RAS/MAPK and PI3K/AKT signaling pathways[53]; an effect that would be expected to be diminished with relatively lower expression in responders compared to non-responders. **MPP3** is member of the family of membrane-associated proteins that interact with the cytoskeleton and regulate cell proliferation, signaling pathways, and intracellular junctions. PI3K can be activated by forming a complex with MAGuK-family proteins **MPP3**[54]. Studies exploring the activation of PI3K/AKT/mTOR signaling in HPV-induced cancers find that erlotinib can induce growth delay of xenografted HPV-containing cervical carcinoma cells [55]. Under expression of **MPP3** in BATTLE responders could contribute to reduced activation of PI3K and enhanced erlotinib efficacy. A detailed discussion of this analysis appears in **S3 Text.**

**Fig 5:**
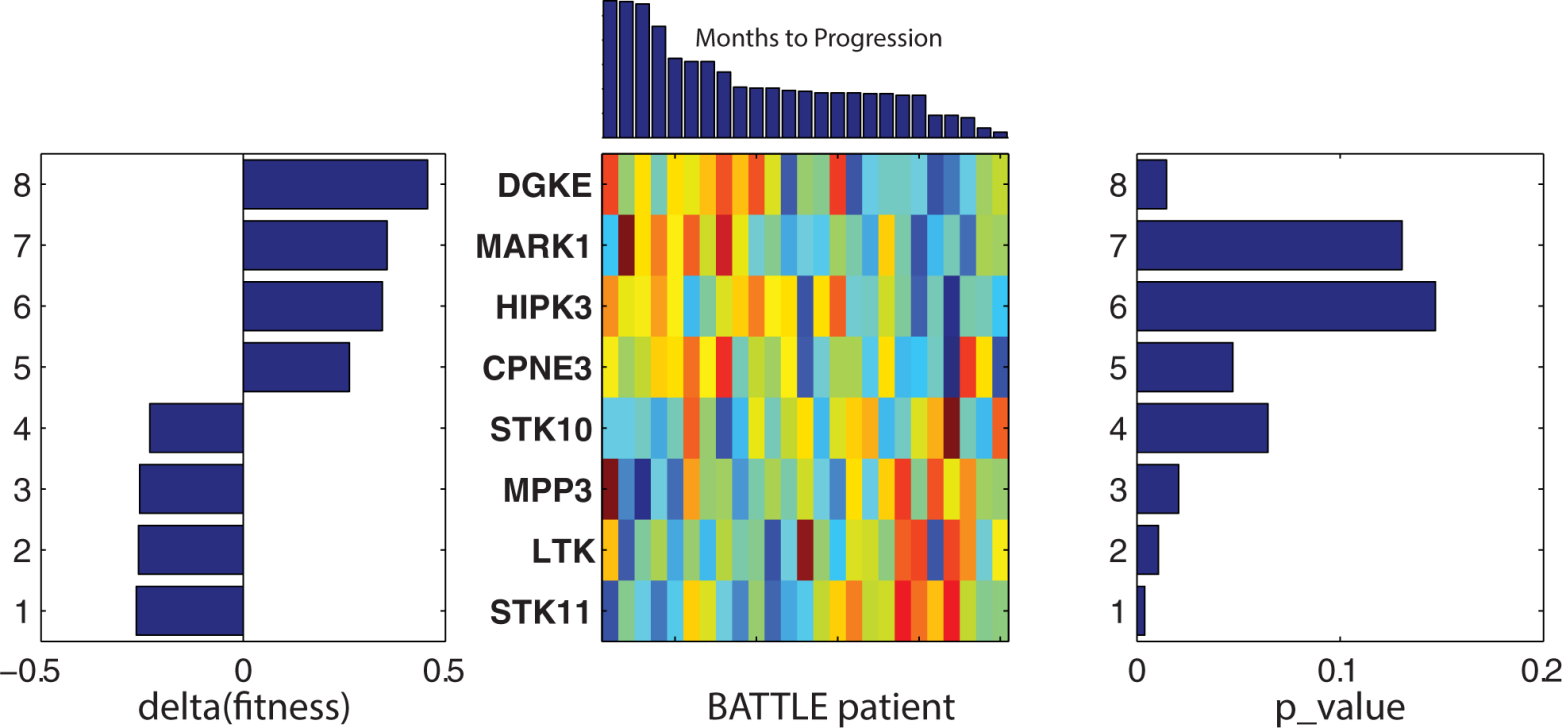
Erlotinib meta-cluster (rows 15–23): The middle panel displays the expression profiles for the genes in meta-clade rows 15–23. Expressions are ordered vertically according to their contribution to the total pathway fitness score, delta(fitness), which is displayed in the left panel. The right panel displays the statistical significance (p_value) for either the t-test comparing the upper and lower 20^th^ percentiles of patient responses or the correlation of gene expression to Months to Progression (shown above the middle panel).

**Table 1:** Summary of the potential erlotinib biomarker genes identified using pathway fitness scores. Column 1 identifies the meta-cluster as viewed in **Fig 4**. Column 2 list whether the pathway fitness score is positive (+) or negative (-). Column 3 lists the gene. Column 4 lists the differential gene expression comparing responders(R) to non-responders(N). Over expression in responders is indicated by a +/-, and vice versa for over expression in non-responders. Column 5 lists the putative MOAs.

Meta-cluster	H	Gene	Expression (R/N)	Putative MOA
Rows 15–23,24–32,33–38	+	STK11, STK10	-/+	ATP homeostasis and increased ROS [35].
Rows 15–23	+	MPP3	-/+	cytoskeleton, cell proliferation, signaling pathways, and intracellular junctions.
Rows 15–23	+	LTK	-/+	promote growth and survival via RAS/MAPK
Rows 15–23,24–32	+	HIPK3	+/-	transcriptional regulation, signal transduction, and regulation of protein stability[36]
Rows 15–23,24–32	+	MARK1	+/-	cell cycle activation and DNA repair[37]
Rows 15–23,24–32	+	DGKE	+/-	regulates protein kinase C (PKC), a family of serine/threonine kinases that has been shown to be involved in EGFR and KRAS signaling[38]
Rows 15–23	+	CPNE3	+/-	ERBB2-mediated tumor cell migration [39]
Rows 24–32	+	SMARCA5	+/-	helicase and ATPase activities[40]
Rows 24–32	+	RUVBL1	+/-	helicase DNA-binding partners involved in EGFR-mediated transcriptional activation[41]
Rows 33–38	+	ANAPC2	-/+	ubiquitin ligase essential for mitotic progression[42]
Rows 33–38	+	GCLM	-/+	ER stress response[43]
Rows 33–38	+	WWP1	-/+	E3 ubiquitin ligase that targets HER4 [44]
Rows 33–38	+	MMP16	-/+	extracellular matrix, migration and invasion[45]
Rows 1–14	-	CACNG5	-/+	trafficking and channel gating[46]
Rows 1–14	-	KCNJ3	-/+	cell proliferation[47]
Rows 1–14	-	NOX5	-/+	regulation of redox-dependent processes[48]
Rows 49–55	-	DUSP6	-/+	regulate MAPs[49]
Rows 49–55	-	SBF1	-/+	growth and differentiation[50]

**Fig 6:**
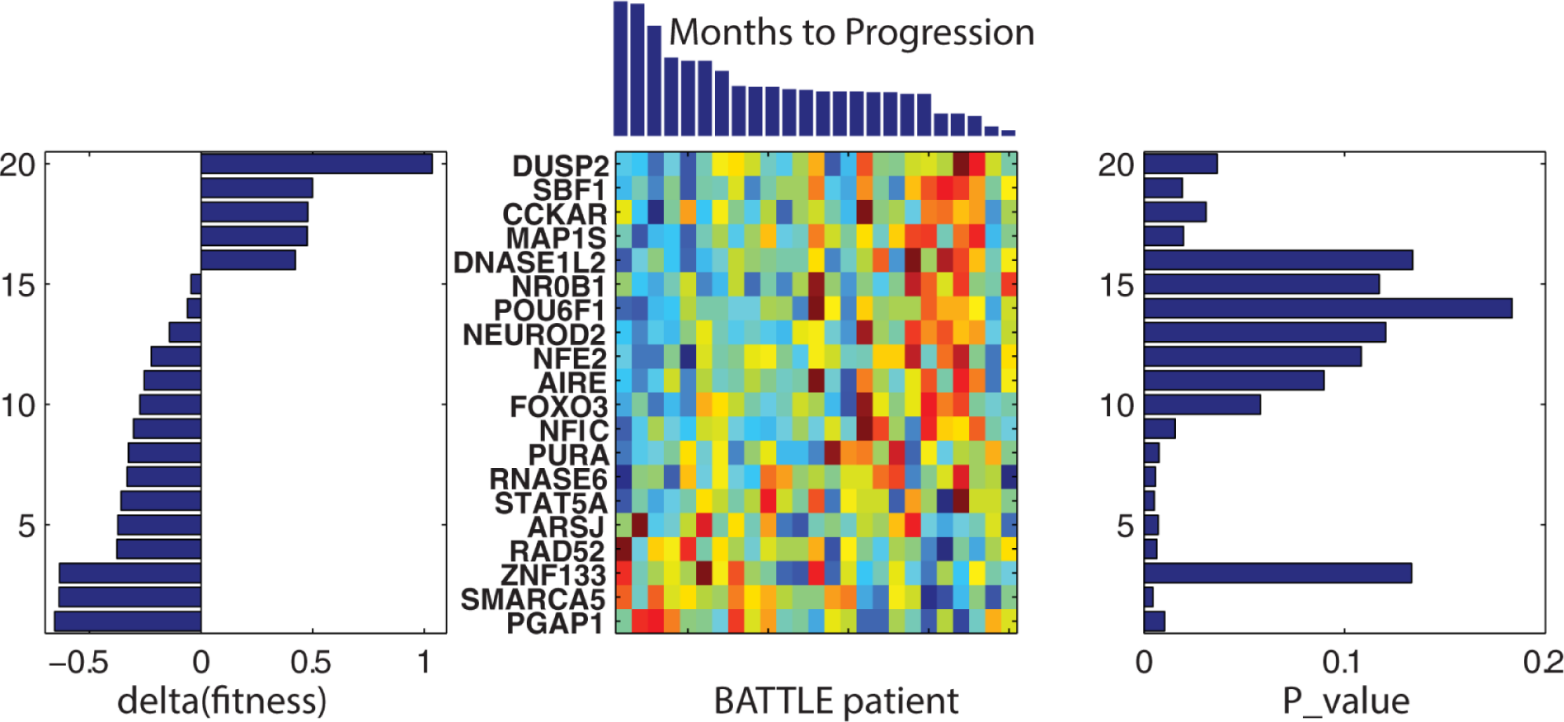
Erlotinib meta-cluster (rows 49–55): The middle panel displays the expression profiles for the genes in meta-cluster(rows 49–55). Expressions are ordered vertically according to their contribution to the total pathway fitness score, delta(fitness), displayed in the left panel. The right panel displays the statistical significance (p_value) for either the t-test comparing the upper and lower 20^th^ percentiles of patient responses or the correlation of gene expression to Months to Progression (shown above the middle panel).

**Fig 6**displays the pathway fitness results for the genes selected in meta-cluster (rows 49–55), with negative fitness scores. This meta-cluster has two genes as top ranked contributors to pathway fitness (DUSP6 and SBF1), both relatively over expressed in non-responders versus responders. **DUSP2** (Dual Specificity Phosphatase 2) is a member of the dual specificity protein phosphatase subfamily that inactivates their target kinases by dephosphorylating both the phosphoserine/threonine and phosphotyrosine residues. They negatively regulate members of the mitogen-activated protein (MAP) kinase superfamily (MAPK/ERK, SAPK/JNK, p38), which are associated with cellular proliferation and differentiation. Their relative under expression in BATTLE responders versus non-responders is consistent with the roles of dual specificity phosphatases in tumor responses to drugs that target Ras/ERK[49]. **SBF1** (SET Binding Factor 1) is a member of the protein-tyrosine phosphatase family. However, the encoded protein does not appear to be a catalytically active phosphatase because it lacks several amino acids in the catalytic pocket. This protein contains a Guanine nucleotide Exchange Factor (GEF) domain which is necessary for growth and differentiation [50]. Over expression of GEFs in erlotinib-resistant cell lines increased NFκB activation in several different types of cancer cells [56].

### Sorafenib—GSEA for 851 genes derived from linear ridge modeling

**Fig 7:**
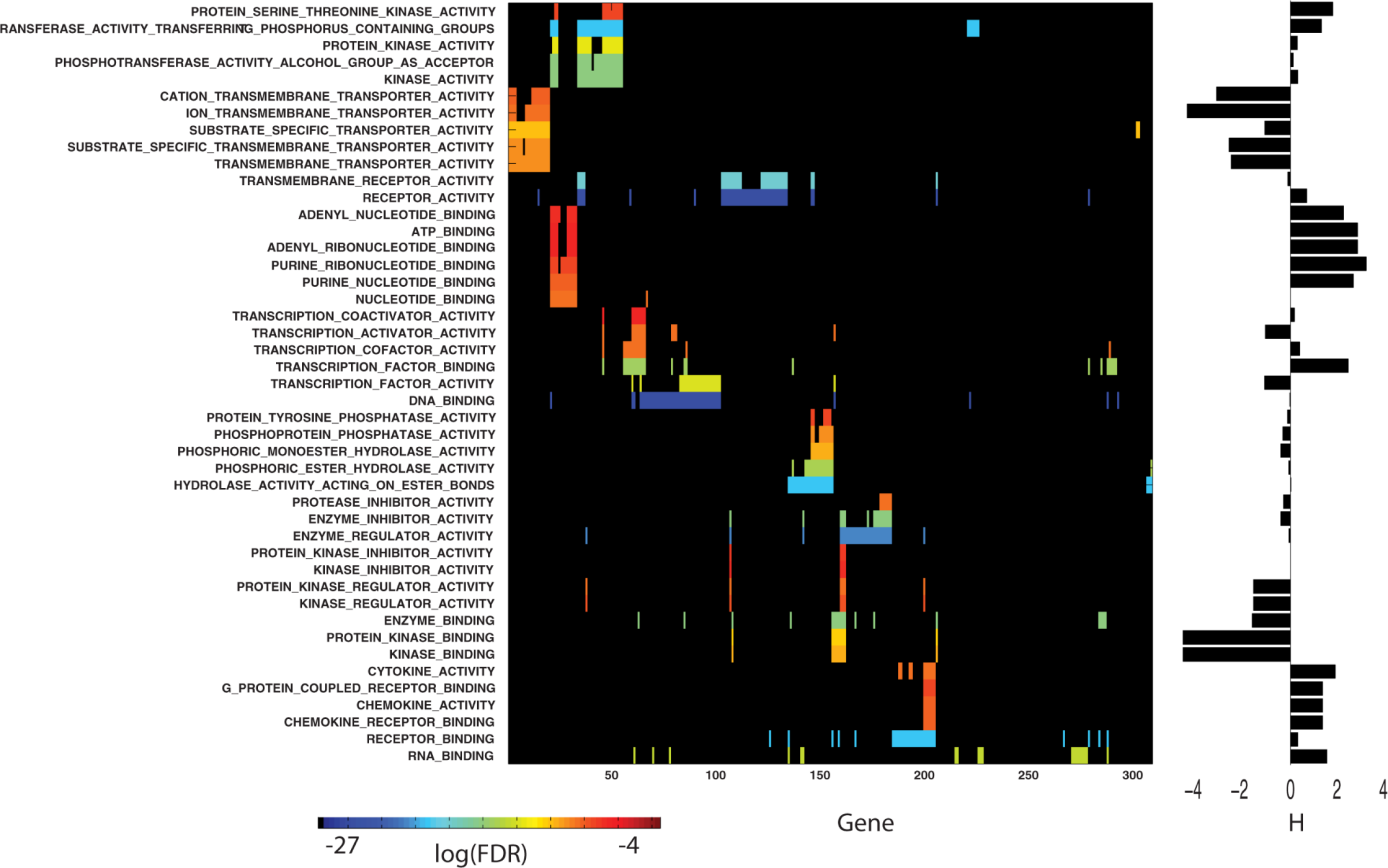
Clustered plot of the topmost significant GSEA pathways and the genes appearing in each pathway. Rows (GSEA Pathways) and columns (one for genes in each pathway, zero otherwise) have been clustered using Minkowski distance metric and Wards linkage. Pathway genes in each row of this clustered plot are colored spectrally by their log(FDR p-val), (blue to red, most to least negative) as listed in **S4 Table**. FDR values were not used for clustering, only the presence or absence of pathway genes. The top 45 GSEA pathways were arbitrarily selected for display to enhance readability of labels. The complete clustered plot for all significant (FDR <0.05) GSEA pathways appears in **S2 Fig**. The pathway fitness scores, H, appear in the vertical bar plot at the right. A minimum of 5 genes are required for a non-zero fitness score.

GSEA finds that 309 of the 851 most frequently appearing genes associated with the 48 linear ridge regressions are found within the GO:molecular function pathways with acceptable FDR q-values. **Fig 7**plots the clustered results (Minkowski distance metric, Wards linkage) for these pathways. As found with erlotinib, these results find better log(FDR q-values) and more shared pathway:gene members in the upper portion of the plot. Meta-clusters with positive fitness scores consist of KINASE related pathways (rows 1–5), RECEPTOR_ACTIVITY and ATP or NUCLEOTIDE_BINDING pathways (rows 11–12,13–18) and CHEMOKINE_ACTIVITY pathways (rows 40–43). Meta-clusters with negative fitness scores consist of TRANSPORTER pathways (rows 6–10) and KINASE_BINDING pathways (rows 35–39). Noteworthy in **Fig 7**are GSEA pathways associated with RECEPTOR_ACTIVITY (rows 11–12) and NUCLEOTIDE_BINDING (rows 13–18) also sharing genes found at the topmost meta-cluster associated with KINASE_ACTIVITY (rows 1–5).

**Table 2**summarizes the pathway-fitness-selected genes for the sorafenib meta-clusters. An illustration of using positive and negative pathway fitness scores for identifying potentially important genes is provided below. An example meta-cluster with positive pathway fitness scores, appearing in meta-cluster (rows 1–5) finds **TYK2, SPHK1**, **EFNA4**, **TRIB1** and **NEK11**. The first three genes are relatively under expressed in sorafenib responders versus non-responders while the latter two genes are relatively over expressed in sorafenib responders versus non-responders. The effects of relative under expression for these genes may be inferred from the literature. **TYK2** is a member of the Janus kinase family which is involved in activating the JAK-STAT (Signal Transducer and Activator of Transcription) signaling pathway and driving cell proliferation [57]. Resistance to sorafenib has been proposed to involve crosstalk between PI3K/AKT and JAK-STAT pathways[58], with literature support for **TYK2** interference with sorafenib efficacy[59]. Under expression of **TYK2** may diminish JAK-STAT’s role in cell proliferation and contribute to enhanced sorafenib efficacy. The pro-apoptotic lipid sphingosine, when phosphorylated by sphingosine kinases (SKs), inclusive of **SPHK1** (Sphingosine Kinase 1), generates the mitogenic lipid sphingosine-1-phosphate. Inhibition of SKs’ activity delays tumor growth in a mouse mammary adenocarcinoma model, suppresses the MAP kinase pathway [60], decreases ERK phosphorylation and is synergistic with sorafenib cytotoxicity [61]. Here, relative **SPHK1** under expression in sorafenib responders when compared to non-responders may parallel these effects and contribute to enhanced sorafenib efficacy. The ephrins (inclusive of **EFNA4**, Eph-Related Receptor Tyrosine Kinase Ligand 4) and EPH-related receptors comprise the largest subfamily of receptor protein-tyrosine kinases and are crucial for migration, repulsion and adhesion during neuronal, vascular and epithelial development. Hypoxia-inducible transcription factor-2alpha in endothelial cells regulates tumor neovascularization through activation of ephrin A1[62]. It has been proposed that hypoxia, induced as a result of the antiangiogenic effects of sustained sorafenib treatment, may be an important factor in sorafenib acquired resistance[63]. Under expression of **EFNA4** may mitigate tumor neovascularization and enhance sorafenib efficacy. Relative gene over expression in BATTLE responders compared to non-responders is observed for NEK11 and TRIB1. **NEK11**, plays an important role in the G2/M checkpoint response to DNA damage [64,65], while **TRIB1** (Tribbles pseudokinase 1) interacts with and regulates activation of MAPK kinases [66]. As potential targets of sorafenib, their relative over expression may offer sites of inhibition that could enhance sorafenib efficacy. A detailed discussion of this analysis appears in **S4 Text.**

**Table 2:** Summary of the potential sorafenib biomarker genes identified using pathway fitness scores. Column 1 identifies the meta-cluster as viewed in **Fig 7**. Column 2 list whether the pathway fitness score is positive (+) or negative (-). Column 3 lists the gene. Column 4 lists the differential gene expression comparing responders(R) to non-responders(N). Over expression in responders is indicated by a +/-, and vice versa for over expression in non-responders. Column 5 lists the putative MOAs.

Meta-cluster	H	Gene	Expression (R/N)	Putative MOA
Rows 1–5	+	TYK2	-/+	signaling cell proliferation[57]
Rows 1–5,13–18	+	SPHK1	-/+	suppresses MAP kinase[60]
Rows 1–5:13–18	+	EFNA4	-/+	migration, repulsion and adhesion[62]
Rows 1–5,13–18	+	NEK11	+/-	response to DNA damage [64,65]
Rows 1–5,13–18	+	TRIB1	+/-	activation of MAP kinases[66]
Rows 13–18	+	RRAGB	+/-	GTPase signal transduction[67]
Rows 13–18	+	BMPR1B	+/-	Serine/threonine protein kinase[68]
Rows 40–43	+	CCL20	+/-	pro-apoptotic cytokine [69]
Rows 6–10	-	SLC5A1	+/-	transport of nutrients and drugs[70]
Rows 6–10	-	SLC1A4	+/-	transport of nutrients and drugs[70]
Rows 6–10	-	SEC61B	-/+	Protein translocation in the ER[71]
Rows 6–10	-	COX4I1	+/-	mitochondrial electron transport[72]
Rows 6–10	-	COX7A1	+/-	mitochondrial electron transport[72]
Rows 6–10	-	KCNK3	+/-	potassium channel proteins[73]
Rows 6–10	-	KCNC3	+/-	potassium channel proteins[73]
Rows 35–39	-	FOXO3	-/+	PI3K/Akt activity[74]
Rows 35–39	-	CDKN2D	-/+	Cyclin-dependent kinase inhibitors[75]
Rows 35–39	-	CDKN2C	-/+	Cyclin-dependent kinase inhibitors[75]

An example of negative fitness scores is meta-cluster (rows 6–10), which consists of TRANSPORTER pathways, mainly comprised of the family of solute carriers (SLC5A6, SLC5A1, SLC13A4, SLC12A1, SLC34A1 and SLC16A7). Top ranked genes contributing to pathway scores include **SLC5A1** and **SLC1A4**, which are over expressed in the responder versus non-responder patients. Over 400 SLC transporter genes have now been identified, representing 55 families, including ion coupled transporters, exchangers and passive transporters located at the plasma membrane or in intracellular organelles. These super families are responsible for mediating the transport of a wide spectrum of substrates, including nutrients and drugs[70]. Cancer cells with enhanced expression of SLC transporters for certain nutritional requirements may provide a growth advantage over normal cells when nutrients become restricted[70]. Sorafenib does not appear to rely on active transport to enter the cell, nor is it a substrate for ABC efflux transporters. Consequently the role of SLC over-expression in sorafenib BATTLE responders does not appear to be related to transporter-mediated alterations of drug influx [76]. A more likely possibility is due to the recent finding that multi-kinase inhibitors also selectively inhibit solute carriers [76,77].

### Erlotinib and sorafenib—Shared genes and GSEA pathways

Fewer than 3% (n = 17 genes) of the genes selected from dual-filtered linear ridge models for erlotinib and sorafenib are common to the GSEA clustered plots in **Figs 4**and **7.** Not surprisingly, approximately half (n = 23, **Table 3**) of the GSEA pathways exist jointly in **Figs 4**and **7**. Shared pathways include KINASE_ACTIVITY, NUCLEOTIDE_BINDING and TRANSPORTER_ACTIVITY, with the latter pathway consistent with preclinical CGP IC_50_ chemo-resistance and poor BATTLE patient responses and the former pathways consistent with preclinical CGP IC_50_ chemo-sensitivity and favorable BATTLE patient responses. These results find potential biomarker genes with divergent roles in compound efficacy. In contrast, a convergent set of pathways appear to be important for compound efficacy, at least for these agents.

**Table 3:** Pathways shared in the GSEA results for erlotinib and sorafenib.

ADENYL_NUCLEOTIDE_BINDING	PHOSPHOTRAFASE_ACT_ALC_GP_AS_ACCPTR
ADENYL_RIBONUCLEOTIDE_BINDING	PROTEIN_KINASE_ACTIVITY
CATION_TRANSMEMBRANE_TRANSPORTER_ACTIVITY	PROTEIN_TYROSINE_PHOSPHATASE_ACTIVITY
DNA_BINDING	PURINE_NUCLEOTIDE_BINDING
HYDROLASE_ACTIVITY_ACTING_ON_ESTER_BONDS	PURINE_RIBONUCLEOTIDE_BINDING
ION_TRANSMEMBRANE_TRANSPORTER_ACTIVITY	RECEPTOR_ACTIVITY
KINASE_ACTIVITY	SUBSTRATE_SPECIFIC_TRMEMBRANE_TRNSPTER_ACTITY
NUCLEOTIDE_BINDING	SUBSTRATE_SPECIFIC_TRANSPORTER_ACTIVITY
PHOSPHOPROTEIN_PHOSPHATASE_ACTIVITY	TRANSCRIPTION_FACTOR_ACTIVITY
PHOSPHORIC_ESTER_HYDROLASE_ACTIVITY	TRANSFERASE_ACTIVITY_TRANSING_PHOSP_GPS
PHOSPHORIC_MONOESTER_HYDROLASE_ACTIVITY	TRANSMEMBRANE_RECEPTOR_ACTIVITY
	TRANSMEMBRANE_TRANSPORTER_ACTIVITY

### Predictive biomarkers

The results above indicate that pathway-genes displayed in **Figs 4**and **7**comprise a potential set of pathway-gene biomarkers (251 for erlotinib and 309 for sorafenib) that link preclinical CGP IC_50_ with BATTLE patient responses. As presented earlier, the number of potential biomarker pathway-genes can be reduced according to their contribution to individual pathway fitness scores, which essentially weights genes within each pathway according to how well their expression profiles match BATTLE patient responses. The differential expressions of these pathway-specific genes have potential roles in compound sensitivity and resistance, and thus cannot be treated independently when assessing their capacity as predictive biomarkers. A collective set of 59 and 51 predictive biomarkers, for erlotinib and sorafenib, respectively, was obtained by selecting GSEA pathway-derived genes in **Figs 4**and **7**with statistical matches (p<0.2) to patient response data and having contributions to pathway fitness scores in the upper and lower 40^th^ percentile of each set of scores. The general aim is to assess how well the subsets of genes contributing the most to pathway fitness scores predict clinical outcome. The results for erlotinib will be presented first.

**Fig 8:**
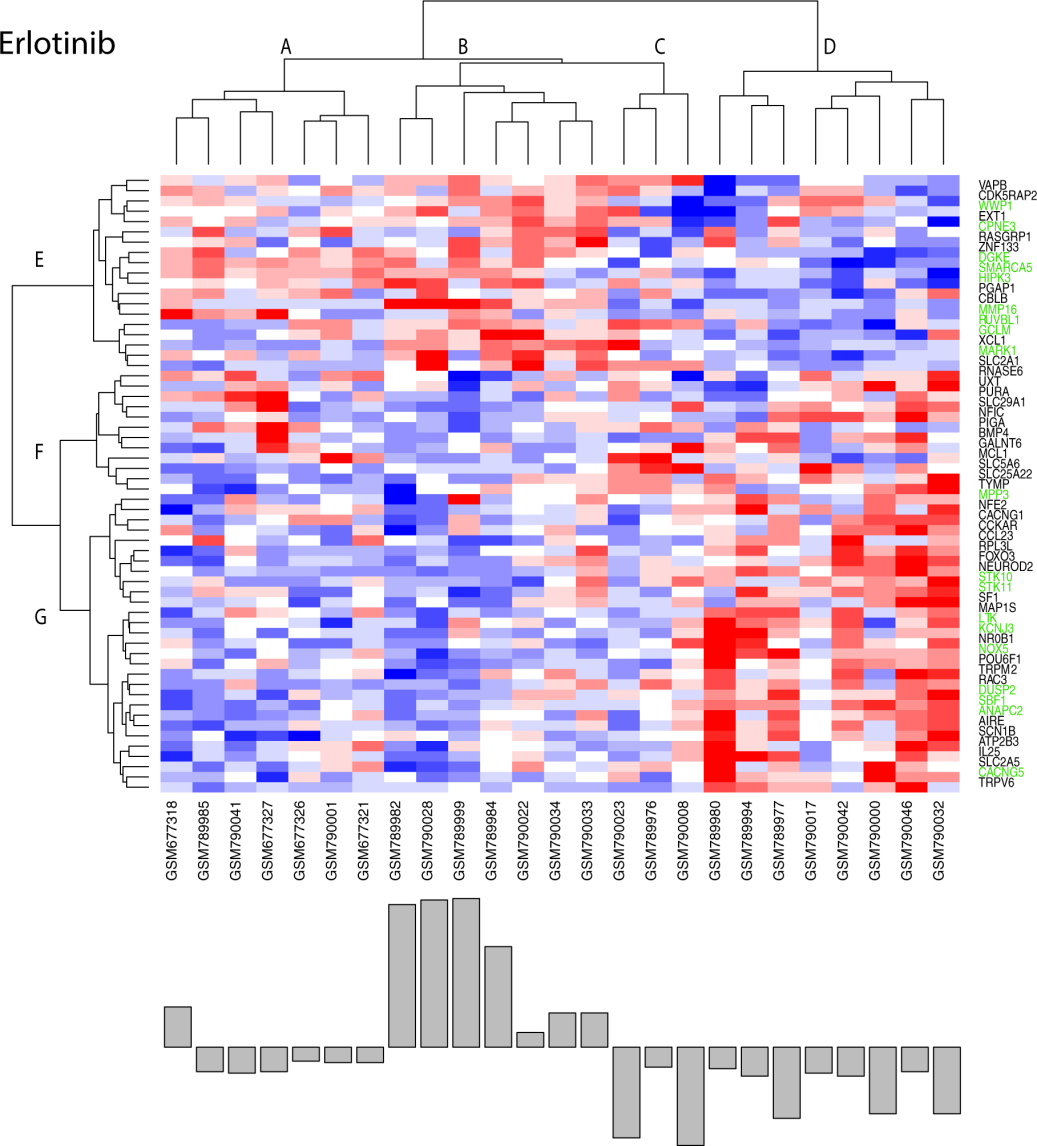
Clustered (Euclidean distance metric, Wards linkage) plot of 59 gene expressions (top panel) selected from genes in the GSEA pathways that have statistical significance (p<0.2) when comparing the top and bottom 20^th^ percentiles of patient responses or have a significant (p<0.2) correlation with the response data and are found in the upper 40^th^ percentile of pathway fitness. Expressions are colored spectrally from blue (under expression) to red (over expression). Bottom panel represents patient responses, ordered according to the clustered genes. Student’s t-test comparing patient response in clades A and B to clades C and D has a significance score of 5.57e-4. Green labels at the right edge identify genes contributing the most to pathway fitness scores (cf. **Table 1**).

**Fig 8**displays the clustered results for the 59 genes that satisfy the above required statistical criteria. Green labels at the right edge identify genes listed in **Table 1**as contributing the most to pathway fitness scores. Row and column clustering of these gene expressions identifies populations of relatively over (red) and under (blue) expressed genes. A bar plot of patient response, ordered according to the independently clustered gene expressions, appears at the bottom of the image to provide a visual indication of gene expressions associated with the better and poorer patient responses. Inspection finds over expression of genes in row clades F and G correspond mostly to non-responders (column clade D), whereas over expression of genes in row clade E corresponds to responder patients (column clades A and B). A Student’s t-test of patient responses in column clades A and B to column clades C and D has a significance score of 5.57e-4. These results provide qualitative support for this set of biomarker pathway-gene expressions as being predictive of erlotinib patient response.

Biomarker pathway-gene expressions for erlotinib can be independently analyzed using singular value decomposition (SVD) to identify which genes contribute the most to the variation in the observed data. SVD is formally derived from the observed data (i.e. gene expressions) and is capable of completely reproducing the data when all principal components (PCs) are used. The eigenvectors associated with these PCs can be used to determine the contribution of each gene to the total variation in the data, referred to as their ‘impact value’. These results find a mutual overlap between genes with the greatest impact values and the genes derived from pathway scores (listed in **S1 Table**). Furthermore, SVD on the complete erlotinib gene subset (cf. **Fig 4)** finds that the pathway-derived genes fall within upper 50^th^ percentile of impact values. These results indicate a qualitative correspondence between genes contributing the most to pathway fitness scores and SVD-derived impact values.

RF calculations were used to determine the clinical prediction errors when using these 59 genes. Class assignments were obtained by calculating RF prediction errors when using different splits of the patient response data. Averaging results from 50 RFs using different seeds finds that a split, where the first 15 patient responses (ordered from greatest to least PFS) are included in the responder class and the last 10 in the non-responder class, produces an average prediction error of 6.8 +- 0.98% for responders and 28.3 +-5.7% for non-responders. This result sets the optimal boundary of class assignment for assessing the role of sample size in prediction errors. For comparison, prediction errors using the 251 genes from GSEA-derived pathways, rather than the 59 derived above, finds that the responder prediction error rate for the optimal split to be slightly poorer (10.1%) while the non-responder prediction error has nearly doubled (47%). Consequently, while relatively good responder prediction errors exist for both gene sets, non-responder prediction errors are considerably higher for the larger gene set. This result supports the likelihood that a failure to accurately predict an unfavorable clinical response may be more difficult when additional gene expressions are considered.

Three additional considerations are important when evaluating RFs predictions. The first explores the robustness of RF predictions when using different data sizes for sampling, validation and testing, while the second provides an indication of the variation in RF error rate due to the random selection of data used for each decision tree, and the third uses area under the receiver-operator curve (AUC_ROC_) to attach a statistical significance to predictions when compared to randomized data. These results find an average RF prediction error (using 50 simulations for each sample size) of 9.8 +- 1.2% and 39.3 +-5.9% for responders (n = 15) and non-responders (n = 10), respectively, for RF predictions using 14, 16, 19 and 21 random samples of master erlotinib dataset (**Fig 8**). The mean and standard deviation associated with these prediction errors grows increasingly large with fewer sample sizes; 8.2+-3.1%, 9.5+-4.9%, 11.0+-8.1%, 11.0+-10.1% for responders and 33.1+-11.0%, 38.0+-13.2%, 39.0+-17.1%, 48.0+-23.3% for non-responders. As expected, the smaller sample sizes diminish the quality of prediction. AUC_ROC_ RF results yield an average of 0.83+-0.09 for these sample sizes, with the lowest AUC_ROC_ (0.78) and the greatest variance (0.12) occurring for the smallest test set (n = 14). The average AUC_ROC_ achieves a statistical significance of p = 3.99e-22 when compared to AUC from randomized input (e.g. AUC = 0.5).

**Fig 9:**
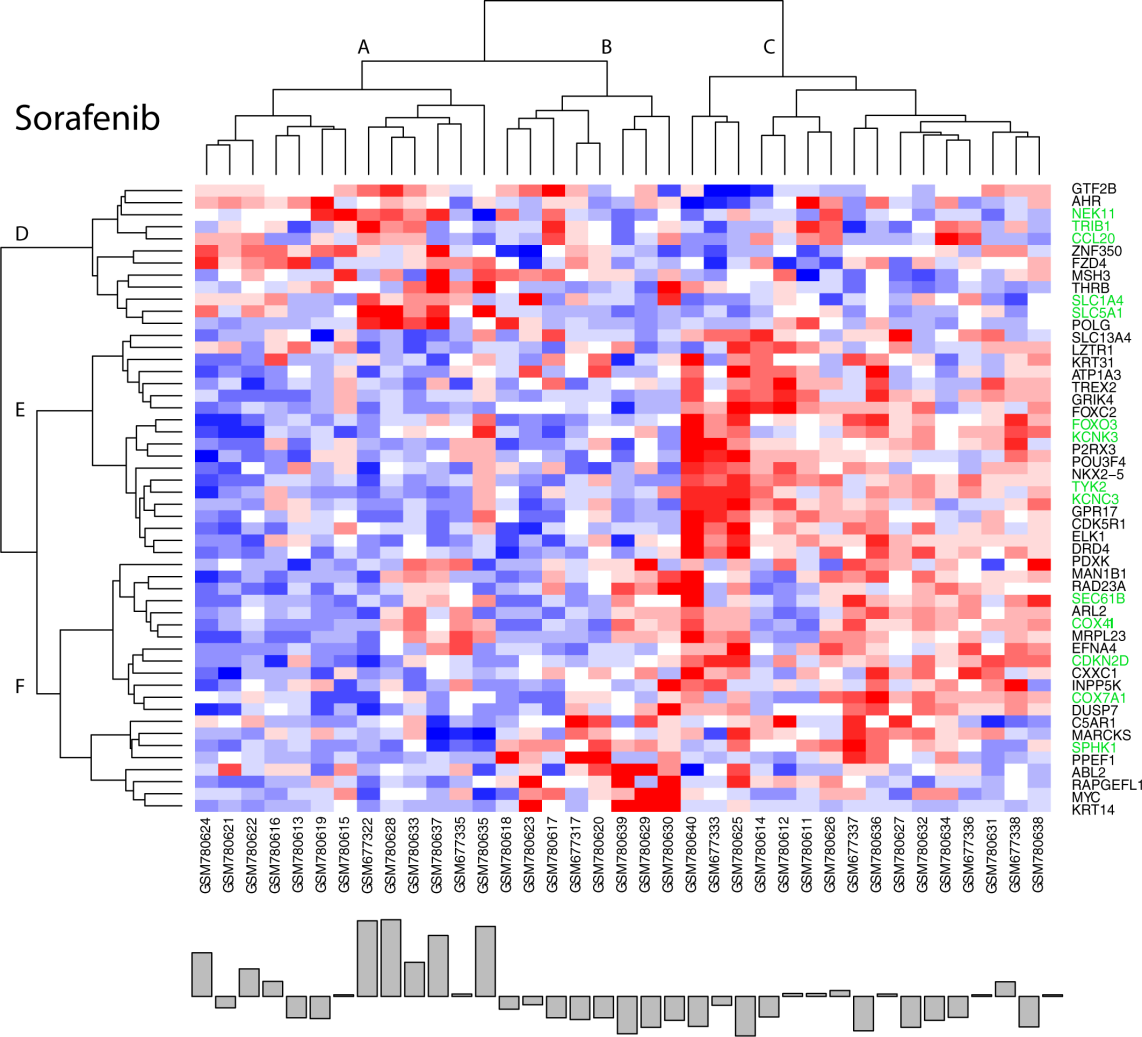
Clustered (Euclidean distance metric, Wards linkage) plot of 51 gene expressions (top panel) selected from genes in the GSEA pathways that have a significant (p<0.2) correlation with the response data. Expressions are colored spectrally from blue (under) to red (over). Bottom panel represents patient responses, ordered according to the clustered genes. A Student’s t-test comparison of the patient responses in clade A compared to clades B and C has a significance of p = 6.70e-5. Green labels at the right edge identify genes contributing the most to pathway fitness scores (cf. **Table 2**).

Similar results are obtained for sorafenib. **Fig 9**clusters the 51 genes (of the 309 linear ridge genes appearing in the sorafenib GSEA pathways in **Fig 7**) to identify populations of relatively over (red) and under (blue) expressed genes. Green labels at the right edge identify genes contributing the greatness to pathway fitness (cf. **Table 2**). A bar plot of patient response, ordered according to the independently clustered gene expressions, appears at the bottom of this image to provide a visual indication of gene expressions associated with the better and worse patient responses. A Student’s t-test comparing the patient responses in column clade A compared to clades B and C has a significance of p = 6.70e-5. SVD of these 51 gene expressions finds the pathway-derived genes listed in **Table 2**fall within the upper 30^th^ percentiles of SVD-derived impact values.

RF results find that using a split with 22 of the most responsive patients in the responder class, with the remaining 15 in the non-responder class produces a minimum prediction error of 17.7 +- 4.7% for responders and 43.6 +- 7.2% for non-responders. A comparison of these prediction errors to results based on the 309 genes from GSEA (**Fig 7**) finds the responder prediction error to be comparable (18.1%), while the non-responder prediction error has increased to 65%. Consequently, as found above with erlotinib, relatively good responder prediction errors exist for both gene sets, yet non-responder prediction errors are considerably higher for the larger gene set, again supporting the likelihood that a failure to achieve a favorable clinical response may be more difficult to predict accurately when additional gene expressions are considered. RF prediction errors for sample sizes of 21, 24, 28 and 31 finds an average of 26.0+-15.5%, 24.4+-13.5%, 20.6+-9.8% and 20.5+-7.2% for responders and 52.8+-11.0%, 54.9+-15.9%, 58.8+-18.3% and 58.3+-17.1% for non-responders, with an overall average of 21.9+-12.9% for responders and 52.4+-16.4% for non-responders. An average AUC_ROC_ of 0.64+-0.10 is found for these 4 samples, with the greatest variance on AUC occurring for the smallest test set (n = 21). The average AUC_ROC_ achieves a statistical significance of p = 6.76e-44 when compared to AUC_ROC_ from randomized input (e.g. AUC = 0.5). For comparison, RF prediction errors based on genes derived from individual meta-clusters (cf. **Figs 4**and **7**) were 5–20% higher when compared to the collective gene set in **Fig 8**. In general, the prediction errors for responders remained reasonably good, while much poorer prediction errors were found for the non-responders.

A number of comparisons were made between of the proposed method for biomarker gene-pathway selection and alternative models using either the complete set of 396 GO:molecular function pathways or the complete set of genes within these pathways that intersect the expressions available for erlotinib (n = 4627) and sorafenib (n = 4850). In brief, pathway fitness scores derived from the complete gene set shared strong correlations with the pathway fitness scores shown in **Figs 4**and **7**(r = 0.843, p = 1.652e-17 for erlotinib and r = 0.752, p = 1.19e-7 for sorafenib). Pathway fitness scores for all 396 pathways found that with the exception of the ATP_BINDING pathway, none of the additional possible GSEA pathways had larger (in absolute value) fitness scores than those found for erlotinib. The sorafenib results for 396 pathways identified GENERAL_RNA_POLYMERASE_II_TRANSCRIPTION_FACTOR_ACTIVITY and STRUCTURE_MOLECULE_ACTIVITY with larger (in absolute value) fitness scores than reported in **Fig 7**. Since neither of these pathways were in the GSEA set (FDR q-value < = 0.05) they were excluded in this analysis. These results indicate the importance of using GSEA for pathway selection. To further amplify the importance of GSEA, pathway fitness scores were obtained for all 396 GO:molecular function pathways using the expanded gene set for erlotinib (n = 4627) and sorafenib (n = 4850). Relatively few (<5) pathways from the existing analysis were found in the best GSEA FDR q-values. These results indicate that the procedures of gene selection using dual filtering of ridge regressions and GSEA of their most frequent genes generates results that are not mimicked when excluding these data mining steps. A detailed discussion of this analysis appears in **S5 Text.**

Expanding on the importance of dual filtering, GSEA was completed for genes selected from the extremes of either log(pval_IC_50_) or log(pval_clinical) (see **Fig 1**). Adjusting thresholds to yield comparable numbers of linear ridge models and completing GSEA for the most frequently occurring genes in these models finds, for erlotinib, 18 GSEA pathways in common to both selection schemes, with 13 of these pathways also found from the dual filtering. The results for sorafenib find 24 GSEA pathways in common to both selection schemes, with 14 of these pathways also found from dual filtering. Notable GSEA pathways excluded from these lists include, for erlotinib; TRANSMEMBRANE_RECEPTOR_PROTEIN_TYROSINE_KINASE_ACTIVITY and the family of TRANSPORT pathways, and for sorafenib; PROTEIN_SERINE_KINASE_ACTIVITY and multiple NUCLEOTIDE_BINDING pathways. In both cases, dual filtering appears to include pathways that are jointly relevant to IC_50_ chemo-responsiveness and patient outcome. These results no not preclude analyses based on pathways derived from genes selected from linear ridge models using either log(pval_IC_50_) or log(pval_clinical), however they suggest that dual-filtering represents a, potentially, superior method for pathway-gene selection.

**Fig 10:**
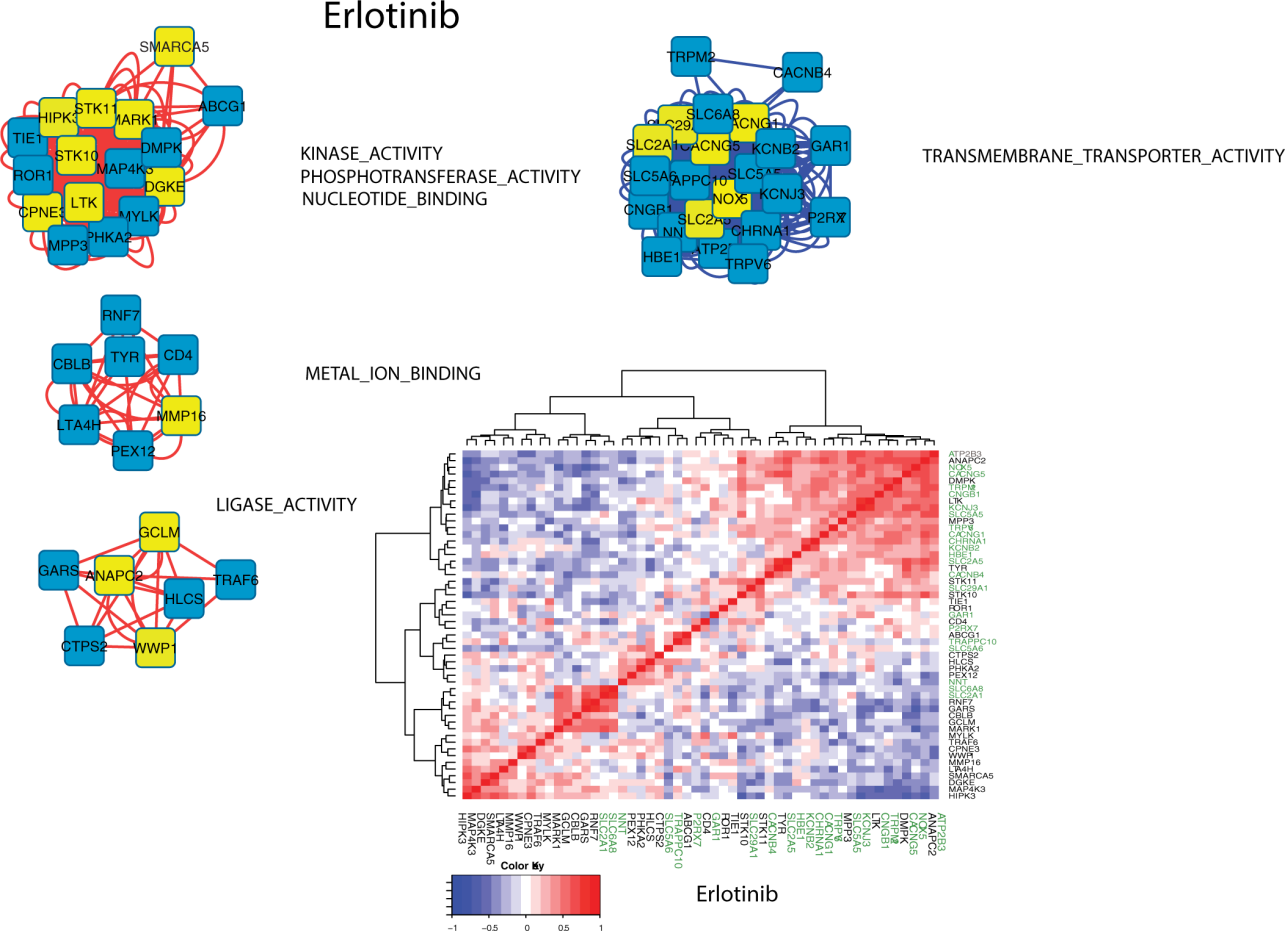
Cytoscape network diagrams using pathway fitness scores as edge weights between pairwise pathway genes (nodes). Results display networks for the upper and lower 20^th^ percentiles of fitness scores. For display purposes, meta-pathway labels are used as abbreviations from those shown in **Fig 4**. Four meta-pathways, comprising fifty-two genes, are identified. Node edges are colored to indicate relative gene expressions between responder and non-responder groups (red: relative over expression, blue: relative under expression). Nodes for genes derived from pathway fitness scores are shown in yellow. The full pathway names for the meta-pathway labels are: KINASE_ACTIVITY/PHOSPHOTRANSFERASE_ACTIVITY/NUCLEOTIDE_BINDING: PROTEIN_KINASE_ACTIVITY PHOSPHOTRANSFERASE_ACTIVITY_ALCOHOL_GROUP_AS_ACCEPTOR TRANSFERASE_ACTIVITY_TRANSFERRING_PHOSPHORUS_CONTAINING_GROUPS ADENYL_NUCLEOTIDE_BINDING ADENYL_RIBONUCLEOTIDE_BINDING NUCLEOTIDE_BINDING PURINE_NUCLEOTIDE_BINDING PURINE_RIBONUCLEOTIDE_BINDING METAL_ION_BINDING: TRANSITION_METAL_ION_BINDING ZINC_ION_BINDING LIGASE_ACTIVITY: ACID_AMINO_ACID_LIGASE_ACTIVITY LIGASE_ACTIVITY TRANSMEMBRANE_TRANSPORTER_ACTIVITY: CATION_TRANSMEMBRANE_TRANSPORTER_ACTIVITY METAL_ION_TRANSMEMBRANE_TRANSPORTER_ACTIVITY SUBSTRATE_SPECIFIC_TRANSMEMBRANE_TRANSPORTER_ACTIVITY SUBSTRATE_SPECIFIC_TRANSPORTER_ACTIVITY TRANSMEMBRANE_TRANSPORTER_ACTIVITY **Lower Right Panel**: Clustered (Euclidean distance metric, Wards linkage) plot of pairwise Pearson correlations (red:+1 blue:-1) for the 52 genes identified in the upper panel. The axis labels for genes associated with pathways having negative fitness scores (TRANSMEMBRANE_TRANSPORTER_ACTIVITY) are highlighted in green. This plot illustrates the concordance of within pathway gene expressions and fitness scores. While individual genes may have expressions that correlate with other pathway genes, it is the cumulative effect of within pathway correlations that leads to the larger fitness scores.

**Fig 10**displays the Cytoscape network derived for erlotinib. Only 52 genes are associated with topmost and bottom most fitness scores of the GSEA pathways for erlotinib. These results indicate that relatively few pathway-genes may serve as potential biomarkers important for identifying favorable and non-favorable patient responses. The lower right plot in **Fig 10**displays the clustered (Euclidean distance metric, Wards linkage) pairwise Pearson correlations for these 52 gene expressions. The axis labels for genes associated pathways having negative fitness scores (TRANSMEMBRANE_TRANSPORTER_ACTIVITY and KINASE_BINDING) are highlighted in green. This plot illustrates the concordance of within pathway gene expressions and fitness scores. While individual genes may have expressions that correlate with other pathway genes, it is the cumulative effect of within pathway correlations that leads to the larger fitness scores.

**Fig 11:**
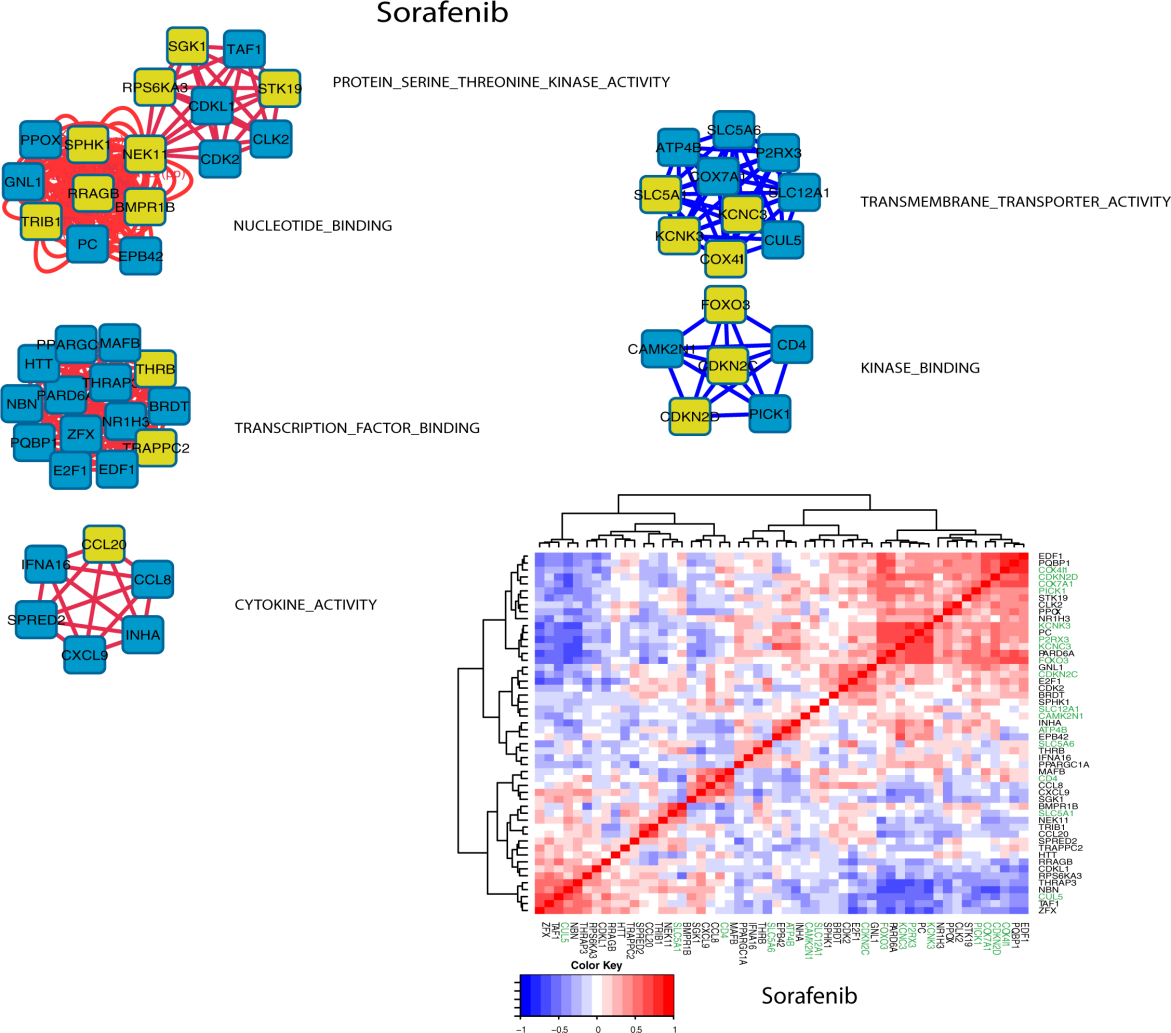
Cytoscape network diagrams using pathway fitness scores as edge weights between pairwise pathway genes (nodes). Results display networks for the upper and lower 20^th^ percentiles of fitness scores. For display purposes, meta-pathways labels, representing abbreviations from those shown in **Fig 7**, are used. Six meta-pathways, comprising 52 genes, are identified for the GSEA genes analyzed. Node edges are colored to indicate relative gene expressions between responder and non-responder groups (red: relative over expression, blue: relative under expression). Nodes for genes derived from pathway fitness are shown in yellow. The full pathway names for the meta-pathway labels are: PROTEIN_SERINE_THREONINE_KINASE_ACTIVITY: PROTEIN_SERINE_THREONINE_KINASE_ACTIVITY NUCLEOTIDE_BINDING: ADENYL_NUCLEOTIDE_BINDING ADENYL_RIBONUCLEOTIDE_BINDING NUCLEOTIDE_BINDING PURINE_NUCLEOTIDE_BINDING PURINE_RIBONUCLEOTIDE_BINDING ATP_BINDING TRANSCRIPTION_FACTOR_BINDING: TRANSCRIPTION_FACTOR_BINDING CYTOKINE_ACTIVITY: CYTOKINE_ACTIVITY TRANSMEMBRANE_TRANSPORTER_ACTIVITY: ION_TRANSMEMBRANE_TRANSPORTER_ACTIVITY KINASE_BINDING: KINASE_BINDING PROTEIN_KINASE_BINDING **Lower Right Panel**: Clustered (Euclidean distance metric, Wards linkage) plot of pairwise Pearson correlations (red:+1 blue:-1) for the 52 genes identified in the upper panel. Genes associated with negative pathway fitness scores (meta-pathways TRANSMEMBRANE_TRANSPORTER_ACTIVITY and KINASE_BINDING are highlighted in green.

**Fig 11**displays the Cytoscape network derived for sorafenib. Only 52 genes are associated with topmost and bottom most fitness scores of the GSEA pathways for sorafenib (having the same number of genes as erlotinib is a coincidence). These results indicate that relatively few pathway-genes may serve as potential biomarkers important for identifying favorable and non-favorable patient responses. The lower right plot in **Fig 11**displays the clustered (Euclidean distance metric, Wards linkage) pairwise Pearson correlations for these 52 gene expressions. The axis labels for genes associated pathways having negative fitness scores (TRANSMEMBRANE_TRANSPORTER_ACTIVITY and KINASE_BINDING) are highlighted in green. This plot illustrates the concordance of within pathway gene expressions and fitness scores. While individual genes may have expressions that correlate with other pathway genes, it is the cumulative effect of within pathway correlations that leads to the larger fitness scores.

## Discussion

It is generally accepted that cancer is a complex disease involving the integration of multiple genomic defects that impact hallmark processes such as cellular proliferation, signaling, DNA repair and replication, and apoptosis[3,78,79]. The converse view, that altered cellular processes (otherwise known as networks or pathways) are the result of individual genomic aberrations, represents an equally attractive idea[4]. A naïve, yet appropriate, extension of this latter view accepts the likelihood that pathways may be vulnerable to cancer-causing perturbations from numerous, and most-likely unrelated, genomic aberrations. The results presented here support this latter view. Application of novel methods of data mining, designed to select for specific phenotypic variations (e.g. preclinical IC_50_ chemo-sensitivity/insensitivity and favorable/unfavorable clinical outcome), identify informative genomic features (gene expression profiles) that collectively reveal shared cellular functions (pathways) and are biologically and clinically predictive. Stratification of these phenotypic variations appears to involve common pathways, many lacking shared genes. This result is consistent with Waddington’s theory of genetic canalization (robustness)[80,81], where pathways sharing common biological functions may lack shared genomic features, yet have an impact on phenotypic variations in, for example, preclinical IC_50_ and clinical outcome. An equally interesting consequence of canalization’s role in providing a strong defense against genomic defects is the likelihood that pathways sharing biological functions may offer additional opportunities for therapeutic attack. Consequently, drugs that impact any given pathway also impact neighboring pathways that share common biological functions; an effect that may contribute to enhanced efficacy or unwanted side-effects. Thus, it is no surprise that an analysis of preclinical and clinical data from these two putative TKIs finds nearly 50% of their indicated GSEA pathways to be in common. Although few genes are shared between these common pathways (**Table 3**) many have been noted as important for TKIs. Examples include;

- ROR1 (Receptor Tyrosine Kinase-Like Orphan Receptor 1) is involved in signaling by GCPR and ERK, has GO annotations for transferring phosphorus-containing groups and protein tyrosine kinase activity (http://www.genecards.org) and is an important paralog of this gene is ALK. Increased expression of ROR1 is associated with B-cell chronic lymphocytic leukemia and is constitutively phosphorylated in chronic lymphocytic *leukemia* (CLL) [82,83] and Glioblastoma multiforme (GBM)[84].
- FOXO3 (forkhead box 3) expression plays a critical role in EGFR tyrosine kinase inhibitor-induced BIM expression and apoptosis[85,86].
- FZD4 (frizzled family receptor 4), a receptor for Wnt proteins, is a mediator of *ERG* oncogene–induced Wnt signaling and epithelial-to-mesenchymal transition in human prostate cancer cells[87]. The Wnt/β-catenin pathway is well implicated in multiple tumors[88].
- WWP1 (WW Domain Containing E3 Ubiquitin Protein Ligase 1) is a Protein Coding gene. Among its related pathways are Signaling by GPCR and the Immune System. GO annotations related to this gene include ligase activity and ubiquitin-protein transferase activity.

Transitioning these potentially important biomarker genes into biomarker pathways finds support in the existing literature. For example, epithelial-mesenchymal transition (EMT) genes have been proposed as biomarkers for deciphering survival and drug responses of cancer patients [89] via a set of 315 EMT biomarker genes as indicators of patient response. Using these biomarker genes, GSEA identified 44 GO:Molecular Function pathways (FDR q-value <0.05). Twenty of these pathways are common to the 24 pathways shared (**Table 3**) in the GSEA results for erlotinib (**Fig 4**) and sorafenib (**Fig 7**). Identifying convergent pathways from divergent genes supports a role for GSEA pathways, in addition to their constituent pathway genes, as joint pathway-gene biomarkers of patient response.

It is important to emphasize that the results presented here cannot be regarded as an appropriate ‘validation’ of the models developed in this analysis. More correctly, the typical model validation process, whereby a proposed model’s ability to predict a response without using validation data, has been incorporated into the modeling process. Consequently, proposing a model that *a priori* includes validation data, then assessing how well validation data can be predicted, represents circular reasoning. However, building models in this manner provides a means to quantify how well such a model can predict response data, then, with an acceptable outcome, critically examine the modeling components (e.g. genes and pathways) for relevance to compound efficacy. Failure to achieve acceptable outcomes, even with the use of validation data in the modeling process, would considerably undermine efforts to link preclinical and clinical data. Acceptable outcomes, however, may provide a foundation for strengthening models with additionally available clinical data and proposing modifications for improvement. Thus, the results generated in this analysis do not represent a ‘validation’ of this modeling effort, rather a means to identify consistent themes that link preclinical and clinical data.

**Fig 12:**
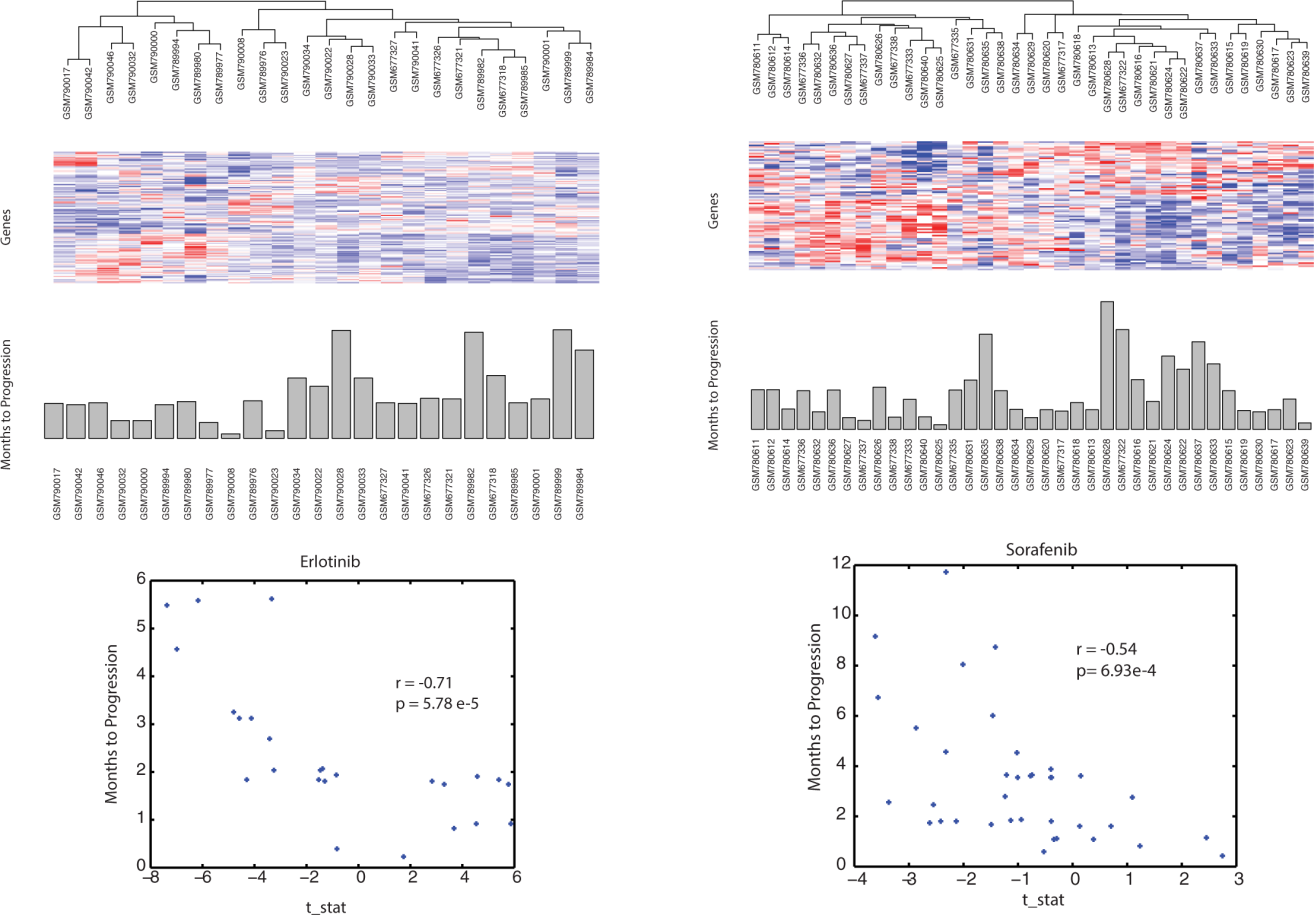
Results for selecting n = 127(erlotinib; left half) and n = 81(sorafenib; right half) patient-derived gene expressions using pathway scores. Top panel for each drug displays the dendrogram from the clustered organization (correlation distance metric, Wards linkage) of gene expressions (second panel). Third panel displays the Months to Progression for patients organized according to the dendrogram in the top panel. Fourth panel display the correlation of the t_stat from a t-test comparing the gene expressions from pathways with large positive H scores to gene expressions from pathways with large negative H scores to Months to Progression.

Extending these biomarker pathway-genes into predictions of patient response remains a significant challenge. Notably, the prediction errors would need to be substantially lowered to enhance use in a clinical setting. Improving non-responsive patient prediction would appear to be an immediate goal. Another consideration involves practical implementation. Ideally, each new patient’s gene expression profiles would be appended to the existing sets of 25 and 37 BATTLE patients, then re-analyzed, as above, with assessment of RF prediction errors. While this effort will be important for building the database needed for modeling patient response, this does not address each patient separately. An alternative approach towards patient-specific assessments is suggested from the results reported herein. Recall that pathways with positive and negative fitness scores are associated with clinical responders and non-responders, respectively. As a consequence, a simple t-test can be constructed for each patient by comparing two pooled sets of gene expressions; one from genes contributing the greatest to H>0 pathway scores (pooled_positive_expressions) and the other from genes contributing the greatest to H<0 pathway scores (pooled_negative_expressions). **Fig 12**summarizes this process for erlotinib and sorafenib. The left (erlotinib) and right (sorafenib) portions of this figure plot, in the upper panel; the dendrogram for clustering the collective sets of gene expressions (i.e. pooled_positive_expressions and pooled_negative_expressions), in the second panel; the clustered gene expressions (n = 127 for erlotinib and 81 for sorafenib) and in the third panel; the Months to Progression, ordered according to the top dendrogram. The fourth panel plots the t_statistic comparing each patient’s pooled_negative_expressions to pooled_positive_expressions versus Months to Progression. Pearson correlations in this fourth panel yield significant results; erlotinib (r = -0.71, p<5.78e-5, n = 127 genes) and sorafenib (r = -0.54, p = 6.9e-4, n = 81 genes). These results support the potential use of a relatively small number of pathway-genes, pooled according to their appearance in selected sets of pathways (i.e. large absolute(H)), as a potential means to identify responder from non-responder BATTLE patients. This strategy, albeit highly speculative, provides a practical approach for using pathway-gene biomarkers as possible indicators of individual patient responses to erlotinib and sorafenib. The feasibility of this speculative approach can be tested with additional clinical data.

## Conclusions

These results offer multiple, potential criteria for predicting a patient’s therapeutic response. Stressing that these criteria follow from i) an analysis using linear ridge modeled results that ii) have been dually filtered using thresholds for model fits of existing preclinical IC_50_ and clinical data, then iii) further filtered for existence within GSEA GO:molecular function pathways, and iv) reduced according to their contribution to pathway fitness scores. Differential gene expressions of these filtered genes yield models with an optimal RF prediction error below 22% for patient responders receiving either sorafenib or erlotinib. Optimal RF prediction errors for non-responders are nearly twice those found for responders. While an explanation for this difference cannot be addressed here, this result may be an indication than the opportunities for a compound failing may greatly exceed those for succeeding, and by extension, more difficult to predict. For example, the results found here clearly support a role for TRANSPORT in non-responders. This type of activity would include the numerous resistance mechanisms involving in the cellular export of a drug. Collectively, these results suggest potentially powerful roles for biomarker pathway-genes when predicting clinical responses from preclinical data.

## Supporting information

S1 Table Erlotinib: Performance statistics for the 53 accepted regression training models. (DOC)

S2 Table Sorafenib: Performance statistics for the 48 accepted regression training models. (DOC)

S3 Table Erlotinib: Complete list of GSEA pathways for erlotinib. (DOC)

S4 Table Sorafenib: Complete list of GSEA pathways for sorafenib. (DOC)

S1 Fig Erlotinib: Clustered plot of all 100 GSEA pathway-gene associations. (DOC)

S2 Fig Sorafenib: Clustered plot of all 100 GSEA pathway-gene associations. (DOC)

S1 Text Erlotinib—Statistical analysis of potential biomarker genes. (DOC)

S2 Text Sorafenib—Statistical analysis of potential biomarker genes. (DOC)

S3 Text Erlotinib—Pathway fitness identified genes. (DOC)

S4 Text Sorafenib—Pathway fitness identified genes. (DOC)

S5 Text Exploring pathway fitness scores across different gene sets. (DOC)
