# Supplementary material for: A data mining approach for identifying pathway-gene biomarkers for predicting clinical outcome: A case study of erlotinib and sorafenib
Source: PLoS One. 2017 Aug 8;12(8):e0181991. doi: 10.1371/journal.pone.0181991 (PMC5549706; doi:10.1371/journal.pone.0181991)
Supplement: S1 Table — (DOC) [file pone.0181991.s001.doc]

**S1 Table.** Erlotinib

Summary of linear ridge results for the 53 models selected for analysis. Column labels represent 1) model id, 2) r for model fit to clinical outcome, 3) log of Pearson correlation coefficient for model fit to observed clinical outcome, 4) positive predictive value of fit, 5) negative predictive value of fit, 6) R^2^ for model predicted IC_50_ to model averaged gene expressions, 7) log of Pearson correlation coefficient for predicted IC_50_ to model averaged gene expressions. Last row represents average values.

| Id # | r | log(pval_clin) | ppv | npv | r | log(pval_IC_50_) |
| --- | --- | --- | --- | --- | --- | --- |
| 1 | - 0.587 | -6.205 | 0.444 | 0.750 | 0.726 | -12.131 |
| 2 | - 0.581 | -6.072 | 0.555 | 0.812 | 0.786 | -15.156 |
| 3 | - 0.620 | -6.982 | 0.454 | 0.785 | 0.714 | -11.580 |
| 4 | - 0.590 | -6.263 | 0.416 | 0.769 | 0.702 | -11.120 |
| 5 | - 0.585 | -6.153 | 0.500 | 0.800 | 0.804 | -16.292 |
| 6 | - 0.634 | -7.335 | 0.500 | 0.764 | 0.846 | -19.417 |
| 7 | - 0.679 | -8.588 | 0.500 | 0.764 | 0.770 | -14.290 |
| 8 | - 0.668 | -8.267 | 0.625 | 0.823 | 0.723 | -11.983 |
| 9 | - 0.608 | -6.693 | 0.500 | 0.764 | 0.744 | -12.924 |
| 10 | - 0.588 | -6.239 | 0.545 | 0.857 | 0.792 | -15.519 |
| 11 | - 0.599 | -6.484 | 0.500 | 0.846 | 0.799 | -15.992 |
| 12 | - 0.619 | -6.944 | 0.500 | 0.764 | 0.795 | -15.699 |
| 13 | - 0.618 | -6.928 | 0.500 | 0.764 | 0.710 | -11.421 |
| 14 | - 0.633 | -7.290 | 0.571 | 0.777 | 0.818 | -17.209 |
| 15 | - 0.646 | -7.648 | 0.555 | 0.812 | 0.756 | -13.559 |
| 16 | - 0.617 | -6.906 | 0.625 | 0.823 | 0.708 | -11.341 |
| 17 | - 0.634 | -7.314 | 0.500 | 0.764 | 0.746 | -13.043 |
| 18 | - 0.637 | -7.401 | 0.555 | 0.812 | 0.720 | -11.856 |
| 19 | - 0.587 | -6.201 | 0.555 | 0.812 | 0.719 | -11.819 |
| 20 | - 0.579 | -6.028 | 0.555 | 0.812 | 0.725 | -12.082 |
| 21 | - 0.589 | -6.241 | 0.555 | 0.812 | 0.744 | -12.953 |
| 22 | - 0.599 | -6.476 | 0.500 | 0.800 | 0.725 | -12.064 |
| 23 | - 0.605 | -6.620 | 0.500 | 0.764 | 0.771 | -14.298 |
| 24 | - 0.663 | -8.114 | 0.500 | 0.764 | 0.786 | -15.147 |
| 25 | - 0.601 | -6.512 | 0.600 | 0.866 | 0.731 | -12.331 |
| 26 | - 0.594 | -6.368 | 0.500 | 0.736 | 0.741 | -12.792 |
| 27 | - 0.581 | -6.073 | 0.571 | 0.777 | 0.746 | -13.020 |
| 28 | - 0.624 | -7.065 | 0.500 | 0.764 | 0.754 | -13.426 |
| 29 | -0.579 | -6.022 | 0.555 | 0.812 | 0.737 | -12.624 |
| 30 | - 0.631 | -7.237 | 0.500 | 0.764 | 0.738 | -12.669 |
| 31 | - 0.728 | -10.230 | 0.666 | 0.875 | 0.702 | -11.093 |
| 32 | - 0.606 | -6.642 | 0.555 | 0.812 | 0.805 | -16.331 |
| 33 | - 0.589 | -6.242 | 0.500 | 0.764 | 0.810 | -16.688 |
| 34 | - 0.598 | -6.445 | 0.500 | 0.800 | 0.701 | -11.080 |
| 35 | - 0.599 | -6.465 | 0.555 | 0.812 | 0.825 | -17.762 |
| 36 | - 0.613 | -6.811 | 0.500 | 0.764 | 0.730 | -12.271 |
| 37 | - 0.675 | -8.457 | 0.666 | 0.875 | 0.753 | -13.392 |
| 38 | - 0.620 | -6.978 | 0.555 | 0.812 | 0.717 | -11.736 |
| 39 | - 0.663 | -8.124 | 0.500 | 0.764 | 0.804 | -16.287 |
| 40 | - 0.590 | -6.263 | 0.636 | 0.928 | 0.757 | -13.609 |
| 41 | - 0.580 | -6.054 | 0.714 | 0.833 | 0.745 | -12.983 |
| 42 | - 0.649 | -7.710 | 0.555 | 0.812 | 0.800 | -16.026 |
| 43 | - 0.603 | -6.573 | 0.500 | 0.800 | 0.818 | -17.222 |
| 44 | - 0.613 | -6.794 | 0.500 | 0.764 | 0.714 | -11.603 |
| 45 | - 0.621 | -7.008 | 0.500 | 0.764 | 0.731 | -12.341 |
| 46 | - 0.688 | -8.869 | 0.555 | 0.812 | 0.773 | -14.434 |
| 47 | - 0.649 | -7.726 | 0.625 | 0.823 | 0.739 | -12.700 |
| 48 | - 0.591 | -6.291 | 0.500 | 0.764 | 0.717 | -11.716 |
| 49 | - 0.603 | -6.573 | 0.555 | 0.812 | 0.727 | -12.179 |
| 50 | - 0.641 | -7.504 | 0.500 | 0.764 | 0.758 | -13.636 |
| 51 | - 0.638 | -7.443 | 0.500 | 0.800 | 0.795 | -15.711 |
| 52 | - 0.619 | -6.952 | 0.545 | 0.857 | 0.705 | -11.221 |
| 53 | - 0.608 | -6.695 | 0.500 | 0.800 | 0.723 | -11.983 |
| Average | -0.641 | -6.972 | 0.540 | 0.790 | 0.754 | -13.581 |
