# Supplementary material for: A data mining approach for identifying pathway-gene biomarkers for predicting clinical outcome: A case study of erlotinib and sorafenib
Source: PLoS One. 2017 Aug 8;12(8):e0181991. doi: 10.1371/journal.pone.0181991 (PMC5549706; doi:10.1371/journal.pone.0181991)
Supplement: S3 Table — (DOC) [file pone.0181991.s003.doc]

**S3 Table.** Erlotinib

GSEA results for 741 genes identified for erlotinib.

| Gene_Set_Name | Description | #_Genes_in_Overlap_(k) | FDR q-value |
| --- | --- | --- | --- |
| RECEPTOR_ACTIVITY | GO:0004872 | 39 | 1.22E-13 |
| SUBSTRATE_SPECIFIC_TRANSPORTER_ACTIVITY | GO:0022892 | 30 | 2.99E-12 |
| SUBSTRATE_SPECIFIC_TRANSMEMBRANE_TRANSPORTER_ACTIVITY | GO:0022891 | 27 | 2.06E-11 |
| TRANSMEMBRANE_TRANSPORTER_ACTIVITY | GO:0022857 | 28 | 2.83E-11 |
| HYDROLASE_ACTIVITY_ACTING_ON_ESTER_BONDS | GO:0016788 | 21 | 3.82E-09 |
| TRANSMEMBRANE_RECEPTOR_ACTIVITY | GO:0004888 | 26 | 6.84E-09 |
| ION_TRANSMEMBRANE_TRANSPORTER_ACTIVITY | GO:0015075 | 21 | 6.85E-09 |
| CATION_TRANSMEMBRANE_TRANSPORTER_ACTIVITY | GO:0008324 | 18 | 1.45E-08 |
| METAL_ION_TRANSMEMBRANE_TRANSPORTER_ACTIVITY | GO:0046873 | 15 | 1.86E-08 |
| PROTEIN_KINASE_ACTIVITY | GO:0004672 | 19 | 2.51E-07 |
| TRANSMEMBRANE_RECEPTOR_PROTEIN_TYROSINE_KINASE_ACTIVITY | GO:0004714 | 8 | 3.88E-07 |
| PROTEIN_HETERODIMERIZATION_ACTIVITY | GO:0046982 | 10 | 4.64E-07 |
| CATION_CHANNEL_ACTIVITY | GO:0005261 | 12 | 5.57E-07 |
| TRANSFERASE_ACTIVITY_TRANSFERRING_PHOSPHORUS_CONTAINING_GROUPS | GO:0016772 | 23 | 5.68E-07 |
| ION_BINDING | GO:0043167 | 18 | 6.08E-07 |
| PHOSPHOTRANSFERASE_ACTIVITY_ALCOHOL_GROUP_AS_ACCEPTOR | GO:0016773 | 20 | 6.73E-07 |
| DNA_BINDING | GO:0003677 | 28 | 7.82E-07 |
| KINASE_ACTIVITY | GO:0016301 | 21 | 8.13E-07 |
| ION_CHANNEL_ACTIVITY | GO:0005216 | 13 | 1.01E-06 |
| TRANSMEMBRANE_RECEPTOR_PROTEIN_KINASE_ACTIVITY | GO:0019199 | 8 | 1.52E-06 |
| SUBSTRATE_SPECIFIC_CHANNEL_ACTIVITY | GO:0022838 | 13 | 1.69E-06 |
| PROTEIN_DIMERIZATION_ACTIVITY | GO:0046983 | 14 | 1.77E-06 |
| TRANSITION_METAL_ION_BINDING | GO:0046914 | 11 | 1.95E-06 |
| ZINC_ION_BINDING | GO:0008270 | 10 | 2.01E-06 |
| TRANSFERASE_ACTIVITY_TRANSFERRING_GLYCOSYL_GROUPS | GO:0016757 | 11 | 2.14E-06 |
| CATION_BINDING | GO:0043169 | 15 | 2.31E-06 |
| LIGASE_ACTIVITY | GO:0016874 | 10 | 3.98E-06 |
| PHOSPHOPROTEIN_PHOSPHATASE_ACTIVITY | GO:0004721 | 9 | 6.51E-06 |
| PROTEIN_TYROSINE_KINASE_ACTIVITY | GO:0004713 | 8 | 7.80E-06 |
| PEPTIDE_BINDING | GO:0042277 | 9 | 9.70E-06 |
| LIGASE_ACTIVITY_FORMING_CARBON_NITROGEN_BONDS | GO:0016879 | 8 | 1.39E-05 |
| PHOSPHORIC_MONOESTER_HYDROLASE_ACTIVITY | GO:0016791 | 10 | 1.44E-05 |
| RECEPTOR_BINDING | GO:0005102 | 19 | 1.51E-05 |
| PYROPHOSPHATASE_ACTIVITY | GO:0016462 | 14 | 2.13E-05 |
| HYDROLASE_ACTIVITY_ACTING_ON_ACID_ANHYDRIDES | GO:0016817 | 14 | 2.35E-05 |
| NUCLEASE_ACTIVITY | GO:0004518 | 7 | 2.85E-05 |
| PHOSPHORIC_ESTER_HYDROLASE_ACTIVITY | GO:0042578 | 11 | 4.19E-05 |
| RNA_POLYMERASE_II_TRANSCRIPTION_FACTOR_ACTIVITY | GO:0003702 | 12 | 4.46E-05 |
| NUCLEOSIDE_TRIPHOSPHATASE_ACTIVITY | GO:0017111 | 13 | 4.62E-05 |
| CYTOSKELETAL_PROTEIN_BINDING | GO:0008092 | 11 | 5.95E-05 |
| TRANSCRIPTION_FACTOR_ACTIVITY | GO:0003700 | 17 | 7.57E-05 |
| OXIDOREDUCTASE_ACTIVITY | GO:0016491 | 15 | 8.38E-05 |
| ACETYLGLUCOSAMINYLTRANSFERASE_ACTIVITY | GO:0008375 | 4 | 1.05E-04 |
| NEUROTRANSMITTER_RECEPTOR_ACTIVITY | GO:0030594 | 6 | 1.50E-04 |
| ENZYME_BINDING | GO:0019899 | 11 | 1.63E-04 |
| GATED_CHANNEL_ACTIVITY | GO:0022836 | 9 | 1.70E-04 |
| CALCIUM_CHANNEL_ACTIVITY | GO:0005262 | 5 | 1.76E-04 |
| STRUCTURAL_MOLECULE_ACTIVITY | GO:0005198 | 13 | 1.89E-04 |
| NEUROTRANSMITTER_BINDING | GO:0042165 | 6 | 2.08E-04 |
| PEPTIDE_RECEPTOR_ACTIVITY | GO:0001653 | 6 | 2.08E-04 |
| ACID_AMINO_ACID_LIGASE_ACTIVITY | GO:0016881 | 6 | 3.11E-04 |
| RNA_BINDING | GO:0003723 | 13 | 3.37E-04 |
| NEUROPEPTIDE_RECEPTOR_ACTIVITY | GO:0008188 | 4 | 3.89E-04 |
| BETA_TUBULIN_BINDING | GO:0048487 | 3 | 4.60E-04 |
| NEUROPEPTIDE_BINDING | GO:0042923 | 4 | 4.65E-04 |
| CYTOKINE_ACTIVITY | GO:0005125 | 8 | 5.10E-04 |
| LIPID_BINDING | GO:0008289 | 7 | 5.27E-04 |
| TRANSCRIPTION_FACTOR_BINDING | GO:0008134 | 14 | 5.30E-04 |
| UDP_GLYCOSYLTRANSFERASE_ACTIVITY | GO:0008194 | 5 | 5.58E-04 |
| ENDONUCLEASE_ACTIVITY_GO_0016893 | GO:0016893 | 3 | 6.25E-04 |
| ACTIN_FILAMENT_BINDING | GO:0051015 | 4 | 6.48E-04 |
| RIBONUCLEASE_ACTIVITY | GO:0004540 | 4 | 6.48E-04 |
| VOLTAGE_GATED_CATION_CHANNEL_ACTIVITY | GO:0022843 | 6 | 6.88E-04 |
| PROTEIN_HOMODIMERIZATION_ACTIVITY | GO:0042803 | 8 | 8.01E-04 |
| KINASE_BINDING | GO:0019900 | 6 | 9.41E-04 |
| ENDORIBONUCLEASE_ACTIVITY | GO:0004521 | 3 | 1.06E-03 |
| STEROID_HORMONE_RECEPTOR_ACTIVITY | GO:0003707 | 3 | 1.06E-03 |
| PROTEIN_DOMAIN_SPECIFIC_BINDING | GO:0019904 | 6 | 1.09E-03 |
| GTPASE_ACTIVITY | GO:0003924 | 7 | 1.14E-03 |
| UBIQUITIN_PROTEIN_LIGASE_ACTIVITY | GO:0004842 | 5 | 1.14E-03 |
| VOLTAGE_GATED_CHANNEL_ACTIVITY | GO:0022832 | 6 | 1.17E-03 |
| SMALL_CONJUGATING_PROTEIN_LIGASE_ACTIVITY | GO:0019787 | 5 | 1.37E-03 |
| ACTIN_BINDING | GO:0003779 | 6 | 1.45E-03 |
| IDENTICAL_PROTEIN_BINDING | GO:0042802 | 13 | 1.47E-03 |
| SMALL_PROTEIN_CONJUGATING_ENZYME_ACTIVITY | GO:0008639 | 5 | 1.49E-03 |
| PROTEIN_TYROSINE_PHOSPHATASE_ACTIVITY | GO:0004725 | 5 | 1.63E-03 |
| SH2_DOMAIN_BINDING | GO:0042169 | 3 | 1.64E-03 |
| PROTEIN_SERINE_THREONINE_KINASE_ACTIVITY | GO:0004674 | 10 | 1.95E-03 |
| OXIDOREDUCTASE_ACTIVITY_ACTING_ON_THE_ALDEHYDE_OR_OXO_GROUP_OF_DONORSNAD_OR_NADP_AS_ACCEPTOR | GO:0016620 | 3 | 2.00E-03 |
| TRANSFERASE_ACTIVITY_TRANSFERRING_HEXOSYL_GROUPS | GO:0016758 | 6 | 2.01E-03 |
| SPECIFIC_RNA_POLYMERASE_II_TRANSCRIPTION_FACTOR_ACTIVITY | GO:0003704 | 4 | 2.36E-03 |
| SEQUENCE_SPECIFIC_DNA_BINDING | GO:0043565 | 5 | 2.44E-03 |
| VOLTAGE_GATED_CALCIUM_CHANNEL_ACTIVITY | GO:0005245 | 3 | 2.84E-03 |
| PROTEIN_N_TERMINUS_BINDING | GO:0047485 | 4 | 3.20E-03 |
| NUCLEOTIDE_BINDING | GO:0000166 | 10 | 3.69E-03 |
| TRANSFERASE_ACTIVITY_TRANSFERRING_PENTOSYL_GROUPS | GO:0016763 | 3 | 3.88E-03 |
| CHEMOKINE_ACTIVITY | GO:0008009 | 4 | 4.62E-03 |
| CHEMOKINE_RECEPTOR_BINDING | GO:0042379 | 4 | 5.03E-03 |
| OXIDOREDUCTASE_ACTIVITY_ACTING_ON_THE_ALDEHYDE_OR_OXO_GROUP_OF_DONORS | GO:0016903 | 3 | 5.12E-03 |
| ADENYL_RIBONUCLEOTIDE_BINDING | GO:0032559 | 8 | 5.15E-03 |
| RHODOPSIN_LIKE_RECEPTOR_ACTIVITY | GO:0001584 | 7 | 6.21E-03 |
| PURINE_RIBONUCLEOTIDE_BINDING | GO:0032555 | 9 | 6.37E-03 |
| ADENYL_NUCLEOTIDE_BINDING | GO:0030554 | 8 | 6.37E-03 |
| PHOSPHOLIPID_BINDING | GO:0005543 | 4 | 6.91E-03 |
| TUBULIN_BINDING | GO:0015631 | 4 | 6.91E-03 |
| TRANSCRIPTION_ACTIVATOR_ACTIVITY | GO:0016563 | 8 | 7.30E-03 |
| ENDONUCLEASE_ACTIVITY | GO:0004519 | 3 | 7.37E-03 |
| LIGAND_DEPENDENT_NUCLEAR_RECEPTOR_ACTIVITY | GO:0004879 | 3 | 7.37E-03 |
| OXIDOREDUCTASE_ACTIVITY_ACTING_ON_NADH_OR_NADPH | GO:0016651 | 3 | 7.37E-03 |
| PURINE_NUCLEOTIDE_BINDING | GO:0017076 | 9 | 7.63E-03 |
