# Supplementary material for: A data mining approach for identifying pathway-gene biomarkers for predicting clinical outcome: A case study of erlotinib and sorafenib
Source: PLoS One. 2017 Aug 8;12(8):e0181991. doi: 10.1371/journal.pone.0181991 (PMC5549706; doi:10.1371/journal.pone.0181991)
Supplement: S4 Table — (DOC) [file pone.0181991.s004.doc]

**S4 Table.** Sorafenib

GSEA results for 851 genes identified for sorafenib

| Gene_Set_Name | Description | #_Genes_in_Overlap_(k) | FDR_q-value |
| --- | --- | --- | --- |
| DNA_BINDING | GO:0003677 | 46 | 1.43E-13 |
| RECEPTOR_ACTIVITY | GO:0004872 | 43 | 2.30E-12 |
| ENZYME_REGULATOR_ACTIVITY | GO:0030234 | 29 | 2.85E-10 |
| TRANSFERASE_ACTIVITY_TRANSFERRING_PHOSPHORUS_CONTAINING_GROUPS | GO:0016772 | 32 | 1.50E-09 |
| RECEPTOR_BINDING | GO:0005102 | 30 | 1.50E-09 |
| HYDROLASE_ACTIVITY_ACTING_ON_ESTER_BONDS | GO:0016788 | 25 | 2.22E-09 |
| TRANSMEMBRANE_RECEPTOR_ACTIVITY | GO:0004888 | 30 | 1.33E-08 |
| PHOSPHOTRANSFERASE_ACTIVITY_ALCOHOL_GROUP_AS_ACCEPTOR | GO:0016773 | 25 | 1.56E-07 |
| ENZYME_INHIBITOR_ACTIVITY | GO:0004857 | 15 | 1.95E-07 |
| ENZYME_BINDING | GO:0019899 | 18 | 1.95E-07 |
| KINASE_ACTIVITY | GO:0016301 | 26 | 1.95E-07 |
| TRANSCRIPTION_FACTOR_BINDING | GO:0008134 | 23 | 4.33E-07 |
| PHOSPHORIC_ESTER_HYDROLASE_ACTIVITY | GO:0042578 | 16 | 6.62E-07 |
| RNA_BINDING | GO:0003723 | 20 | 1.98E-06 |
| TRANSCRIPTION_FACTOR_ACTIVITY | GO:0003700 | 23 | 4.64E-06 |
| PROTEIN_KINASE_ACTIVITY | GO:0004672 | 20 | 8.12E-06 |
| PROTEIN_KINASE_BINDING | GO:0019901 | 9 | 3.97E-05 |
| SUBSTRATE_SPECIFIC_TRANSPORTER_ACTIVITY | GO:0022892 | 22 | 7.99E-05 |
| KINASE_BINDING | GO:0019900 | 9 | 1.00E-04 |
| PHOSPHORIC_MONOESTER_HYDROLASE_ACTIVITY | GO:0016791 | 11 | 1.20E-04 |
| NUCLEASE_ACTIVITY | GO:0004518 | 8 | 1.20E-04 |
| PHOSPHOPROTEIN_PHOSPHATASE_ACTIVITY | GO:0004721 | 9 | 2.91E-04 |
| SUBSTRATE_SPECIFIC_TRANSMEMBRANE_TRANSPORTER_ACTIVITY | GO:0022891 | 19 | 3.41E-04 |
| TRANSMEMBRANE_TRANSPORTER_ACTIVITY | GO:0022857 | 20 | 3.41E-04 |
| CATION_BINDING | GO:0043169 | 14 | 6.09E-04 |
| CYTOKINE_ACTIVITY | GO:0005125 | 10 | 6.27E-04 |
| ION_BINDING | GO:0043167 | 16 | 6.55E-04 |
| IDENTICAL_PROTEIN_BINDING | GO:0042802 | 17 | 6.65E-04 |
| ION_TRANSMEMBRANE_TRANSPORTER_ACTIVITY | GO:0015075 | 16 | 7.57E-04 |
| NUCLEOTIDE_BINDING | GO:0000166 | 14 | 8.48E-04 |
| PROTEIN_KINASE_REGULATOR_ACTIVITY | GO:0019887 | 6 | 8.48E-04 |
| LIGASE_ACTIVITY | GO:0016874 | 9 | 8.48E-04 |
| RIBONUCLEASE_ACTIVITY | GO:0004540 | 5 | 9.02E-04 |
| TRANSCRIPTION_COFACTOR_ACTIVITY | GO:0003712 | 14 | 9.16E-04 |
| TRANSCRIPTION_ACTIVATOR_ACTIVITY | GO:0016563 | 12 | 9.16E-04 |
| OXIDOREDUCTASE_ACTIVITY | GO:0016491 | 16 | 9.61E-04 |
| PROTEASE_INHIBITOR_ACTIVITY | GO:0030414 | 6 | 9.63E-04 |
| ENZYME_ACTIVATOR_ACTIVITY | GO:0008047 | 10 | 9.84E-04 |
| GTPASE_REGULATOR_ACTIVITY | GO:0030695 | 10 | 9.84E-04 |
| CHEMOKINE_ACTIVITY | GO:0008009 | 6 | 1.02E-03 |
| CHEMOKINE_RECEPTOR_BINDING | GO:0042379 | 6 | 1.14E-03 |
| PURINE_NUCLEOTIDE_BINDING | GO:0017076 | 13 | 1.30E-03 |
| CATION_TRANSMEMBRANE_TRANSPORTER_ACTIVITY | GO:0008324 | 13 | 1.39E-03 |
| KINASE_REGULATOR_ACTIVITY | GO:0019207 | 6 | 1.56E-03 |
| TRANSITION_METAL_ION_BINDING | GO:0046914 | 9 | 1.69E-03 |
| NUCLEOTIDYLTRANSFERASE_ACTIVITY | GO:0016779 | 6 | 1.69E-03 |
| GENERAL_RNA_POLYMERASE_II_TRANSCRIPTION_FACTOR_ACTIVITY | GO:0016251 | 5 | 2.16E-03 |
| HEMATOPOIETIN_INTERFERON_CLASSD200_DOMAIN_CYTOKINE_RECEPTOR_ACTIVITY | GO:0004896 | 5 | 2.46E-03 |
| PEPTIDE_RECEPTOR_ACTIVITY | GO:0001653 | 6 | 2.93E-03 |
| PROTEIN_TYROSINE_PHOSPHATASE_ACTIVITY | GO:0004725 | 6 | 2.93E-03 |
| PROTEIN_SERINE_THREONINE_KINASE_ACTIVITY | GO:0004674 | 12 | 2.93E-03 |
| PURINE_RIBONUCLEOTIDE_BINDING | GO:0032555 | 12 | 2.93E-03 |
| ACTIVE_TRANSMEMBRANE_TRANSPORTER_ACTIVITY | GO:0022804 | 9 | 2.93E-03 |
| INTERLEUKIN_RECEPTOR_ACTIVITY | GO:0004907 | 4 | 2.99E-03 |
| G_PROTEIN_COUPLED_RECEPTOR_BINDING | GO:0001664 | 6 | 3.05E-03 |
| STRUCTURAL_MOLECULE_ACTIVITY | GO:0005198 | 13 | 3.95E-03 |
| STRUCTURAL_CONSTITUENT_OF_CYTOSKELETON | GO:0005200 | 6 | 3.95E-03 |
| PHOSPHORIC_DIESTER_HYDROLASE_ACTIVITY | GO:0008081 | 5 | 5.08E-03 |
| PROTEIN_KINASE_INHIBITOR_ACTIVITY | GO:0004860 | 4 | 5.67E-03 |
| PEPTIDE_BINDING | GO:0042277 | 7 | 5.92E-03 |
| INTERLEUKIN_BINDING | GO:0019965 | 4 | 6.34E-03 |
| KINASE_INHIBITOR_ACTIVITY | GO:0019210 | 4 | 6.34E-03 |
| ADENYL_NUCLEOTIDE_BINDING | GO:0030554 | 10 | 6.77E-03 |
| SERINE_TYPE_PEPTIDASE_ACTIVITY | GO:0008236 | 5 | 7.94E-03 |
| SERINE_HYDROLASE_ACTIVITY | GO:0017171 | 5 | 8.64E-03 |
| PEPTIDASE_ACTIVITY | GO:0008233 | 10 | 8.70E-03 |
| 3_5_CYCLIC_NUCLEOTIDE_PHOSPHODIESTERASE_ACTIVITY | GO:0004114 | 3 | 8.70E-03 |
| SECONDARY_ACTIVE_TRANSMEMBRANE_TRANSPORTER_ACTIVITY | GO:0015291 | 5 | 9.11E-03 |
| CYTOKINE_BINDING | GO:0019955 | 5 | 9.87E-03 |
| TRANSCRIPTION_COACTIVATOR_ACTIVITY | GO:0003713 | 8 | 1.02E-02 |
| CYCLIC_NUCLEOTIDE_PHOSPHODIESTERASE_ACTIVITY | GO:0004112 | 3 | 1.02E-02 |
| PROTEIN_TRANSPORTER_ACTIVITY | GO:0008565 | 3 | 1.02E-02 |
| RNA_POLYMERASE_II_TRANSCRIPTION_FACTOR_ACTIVITY | GO:0003702 | 10 | 1.02E-02 |
| ATP_BINDING | GO:0005524 | 9 | 1.20E-02 |
| SYMPORTER_ACTIVITY | GO:0015293 | 4 | 1.20E-02 |
| GROWTH_FACTOR_BINDING | GO:0019838 | 4 | 1.32E-02 |
| ACTIN_BINDING | GO:0003779 | 6 | 1.32E-02 |
| ADENYL_RIBONUCLEOTIDE_BINDING | GO:0032559 | 9 | 1.52E-02 |
| RHODOPSIN_LIKE_RECEPTOR_ACTIVITY | GO:0001584 | 8 | 1.56E-02 |
| GROWTH_FACTOR_ACTIVITY | GO:0008083 | 5 | 1.56E-02 |
| MONOVALENT_INORGANIC_CATION_TRANSMEMBRANE_TRANSPORTER_ACTIVITY | GO:0015077 | 4 | 1.56E-02 |
| STRUCTURE_SPECIFIC_DNA_BINDING | GO:0043566 | 5 | 1.65E-02 |
| TRANSFERASE_ACTIVITY_TRANSFERRING_HEXOSYL_GROUPS | GO:0016758 | 6 | 1.69E-02 |
| RECEPTOR_SIGNALING_PROTEIN_ACTIVITY | GO:0005057 | 6 | 1.77E-02 |
| DNA_POLYMERASE_ACTIVITY | GO:0034061 | 3 | 1.83E-02 |
| SEQUENCE_SPECIFIC_DNA_BINDING | GO:0043565 | 5 | 1.83E-02 |
| TRANSFERASE_ACTIVITY_TRANSFERRING_GLYCOSYL_GROUPS | GO:0016757 | 7 | 1.98E-02 |
| GTPASE_ACTIVATOR_ACTIVITY | GO:0005096 | 5 | 2.05E-02 |
| EXONUCLEASE_ACTIVITY | GO:0004527 | 3 | 2.05E-02 |
| ENDOPEPTIDASE_ACTIVITY | GO:0004175 | 7 | 2.42E-02 |
| PROTEIN_TYROSINE_KINASE_ACTIVITY | GO:0004713 | 5 | 2.46E-02 |
| ZINC_ION_BINDING | GO:0008270 | 6 | 2.55E-02 |
| DAMAGED_DNA_BINDING | GO:0003684 | 3 | 2.62E-02 |
| SERINE_TYPE_ENDOPEPTIDASE_ACTIVITY | GO:0004252 | 4 | 2.66E-02 |
| TRANSCRIPTION_REPRESSOR_ACTIVITY | GO:0016564 | 8 | 2.77E-02 |
| TRANSMEMBRANE_RECEPTOR_PROTEIN_TYROSINE_KINASE_ACTIVITY | GO:0004714 | 4 | 3.09E-02 |
| SUBSTRATE_SPECIFIC_CHANNEL_ACTIVITY | GO:0022838 | 8 | 3.15E-02 |
| LIGASE_ACTIVITY_FORMING_CARBON_NITROGEN_BONDS | GO:0016879 | 5 | 3.15E-02 |
| HORMONE_ACTIVITY | GO:0005179 | 4 | 3.25E-02 |
| CYTOSKELETAL_PROTEIN_BINDING | GO:0008092 | 8 | 3.42E-02 |
