# Supplementary material for: A data mining approach for identifying pathway-gene biomarkers for predicting clinical outcome: A case study of erlotinib and sorafenib
Source: PLoS One. 2017 Aug 8;12(8):e0181991. doi: 10.1371/journal.pone.0181991 (PMC5549706; doi:10.1371/journal.pone.0181991)
Supplement: S1 Fig — (DOC) [file pone.0181991.s005.doc]

**S1 Fig.** Erlotinib: Clustered plot of GSEA pathways (y-axis) and the genes (x-axis) appearing in each pathway. Rows (GSEA Pathways) and columns (one’s for genes in each pathway, zeroes otherwise) have been clustered using a Minkowski’s distance metric and Wards linkage. Pathway genes in each row are colored spectrally by their log(FDR q-value), (blue to red, most to least negative) as listed in **S3** **Table**. FDR q-values were not used for clustering, only the presence or absence of pathway genes. The pathway fitness scores, H, appear in the vertical bar plot at the right. A minimum of 5 pathway genes are required for a non-zero fitness score.
