# Supplementary material for: A data mining approach for identifying pathway-gene biomarkers for predicting clinical outcome: A case study of erlotinib and sorafenib
Source: PLoS One. 2017 Aug 8;12(8):e0181991. doi: 10.1371/journal.pone.0181991 (PMC5549706; doi:10.1371/journal.pone.0181991)
Supplement: S2 Fig — (DOC) [file pone.0181991.s006.doc]

**S2 Fig.** Sorafenib: Clustered plot of the significant GSEA pathways and the genes appearing in each pathway. Rows (GSEA Pathways) and columns (one’s for genes in each pathway, zeroes otherwise) have been clustered using Manhattan’s distance metric and Wards linkage. Pathway genes in each row of this clustered plot are colored spectrally by their log(FDR p-val), (blue to red, most to least negative) as listed in **S4 Table**. Coloring these clusters according to their GSEA pathway log(FDR pval) finds the best FDR values in the upper half of the plot to be associated with relatively unique gene members. The bar in the right edge displays the H-scores for GSEA pathways. Inspection of the H-scores for the 100 GSEA pathways (**S4 Table**) finds a general correspondence between larger (absolute) H-scores and better FDR scores. Minimum p-value (5.910e-6) for a split of H-scores occurs at the 45^th^ (pathway 8.87e-4 @ 45). A minimum of 5 genes are required for a non-zero H-score.
