# Supplementary material for: A data mining approach for identifying pathway-gene biomarkers for predicting clinical outcome: A case study of erlotinib and sorafenib
Source: PLoS One. 2017 Aug 8;12(8):e0181991. doi: 10.1371/journal.pone.0181991 (PMC5549706; doi:10.1371/journal.pone.0181991)
Supplement: S1 Text — (DOC) [file pone.0181991.s007.doc]

**S1 Text**

**Erlotinib - statistical analysis of potential biomarker genes**

Over 3k genes appear in the topmost 53 training models, with 741 genes existing in at least two of the 53 training models. These genes constitute a starting set for identifying relationships between gene expressions and responses of preclinical CGP IC_50_ or BATTLE Months to Progression. These gene expressions may also constitute biomarkers that separate erlotinib preclinical chemo-sensitive from chemo-resistant IC_50_, and separate erlotinib treated BATTLE patients that have a clinically favorable, from those that have unfavorable, Months to Progression (referred to hereafter as responders and non-responders, respectively).

Student’s t-tests were employed to establish a traditional perspective for relating gene expressions to preclinical IC_50_ or patient Months to Progression. Analysis of the ~11k gene expressions co-existing in the CGP erlotinib IC_50_ data and the erlotinib treated BATTLE patients finds 1963 genes with statistically significant (p<0.05) expression differences when comparing the top and bottom 30^th^ percentiles of BATTLE clinical responses. A Benjamini-Hochberg(B-H) correction for multiple tests finds only SEMA3D and PLCB1 to satisfy the false discovery threshold of p<=0.05. Completing the same analysis of the erlotinib preclinical CGP IC_50_ data finds 3595 of the 11,844 gene expressions satisfying p<=0.05, with 1622 achieving a B-H threshold of 0.05. Intersection of the 1963 and 3595 genes finds only 12% (n=530) in common (530/(3595-530+1963-530 = 0.118). Collectively, these results yield undesirable conclusions; one due to B-H corrections finding few genes, based on clinical response, and many genes, based on CGP IC_50_, and the other due to the relatively low intersection of genes between each set. Collectively the results derived using traditional statistics do not strongly support a unified set of genes that can are jointly associated with the erlotinib preclinical CGP IC_50_ and BATTLE clinical responses.

Merging the above t-test derived results with the 741 genes identified from linear ridge regressions finds co-existence for 129 of the significantly scoring genes using the BATTLE patient response and 224 of the significantly scoring genes using preclinical CGP IC_50_’s. However, these genes also fail to intersect. Separate analysis of clustered correlation plots for these genes, however, finds evidence in support of GSEA pathways associated with their differential gene expressions for erlotinib preclinical IC_50_ and BATTLE clinical responses. The left panel in **Fig 1** plots the clustered results for the pairwise Pearson correlation coefficients of the subset of 129 genes derived jointly from the BATTLE clinical responses and linear ridge regression, while the right panel displays the same information for the 224 genes derived jointly from the CGP preclinical IC_50_ data and linear ridge regression. The clustered plot in left panel finds a clear pattern of genes over expressed in patients with the best Months to Progression (lower left corner of the left most image: genes 1:31) and genes overexpressed in the patients with the worst Months to Progression (upper right corner of left most image: genes 32:129). GSEA for over expressed genes associated with the best patient responses (i.e. responders) identify GO:Molecular Function Pathways for KINASE ACTIVITY, ATP BINDING and NUCLEOTIDE BINDING. GO: Molecular Function Pathways for genes relatively over expressed in BATTLE patients with the worst outcome (i.e. non-responders) are associated primarily with TRANSPORTER ACTIVITY. The clustered plot in the right panel of **Fig 1** separates over expressed genes associated with CGP preclinical IC_50_ chemo-sensitivity (lower left region genes 1:64) from over expressed genes associated with CGP preclinical IC_50_ chemo-resistance (upper right region genes 65:224). GSEA for the former group of genes finds LIGASE, OXIDOREDUCTASE and DIMERIZATION associated pathways. These genes include EGFR, which functions in ubiquitin protein ligase binding and protein dimerization, and is also consistent with erlotinib targeting the oxidoreductase activity of cytochrome P450 (http://www.drugbank.ca/drugs/DB00530). Genes in the latter group consist primarily of TRANSPORTER pathways. While these results do not identify common genes with expression measures that correspond to preclinical erlotinib IC_50_ and BATTLE clinical responses, sets of pathways are found that associate chemo-resistance and poor patient responses to TRANSPORTER pathways, and chemo-sensitivity and better patient responses to pathways involving known targets of erlotinib.

**Fig 1.** Erlotinib: Left panel; clustered plot of pair-wise gene expression correlations for the 129 genes exhibiting statistical significance (p<=0.05) between the top and bottom 30^th^ percentile (responders versus non-responders, respectively) for erlotinib treated BATTLE patients and co-occurring in the 741 genes derived from linear ridge modeling. Right panel; clustered plot of pair-wise gene expression correlations for the 224 genes exhibiting statistical significance (p<=0.05) between the top and bottom 30^th^ percentile of chemo-response for erlotinib IC_50_ in the CGP database and co-occurring in the 741 genes derived from linear ridge modeling. Color scheme represents the spectrum of correlation values (-1, blue to +1, red). Deeper colors on the left panel indicate a greater separation of correlation values between the BATTLE responders and non-responders, when compared to the CGP IC_50_ chemo-response data.
