# Supplementary material for: A data mining approach for identifying pathway-gene biomarkers for predicting clinical outcome: A case study of erlotinib and sorafenib
Source: PLoS One. 2017 Aug 8;12(8):e0181991. doi: 10.1371/journal.pone.0181991 (PMC5549706; doi:10.1371/journal.pone.0181991)
Supplement: S2 Text — (DOC) [file pone.0181991.s008.doc]

**S2 Text**

**Sorafenib - statistical analysis of potential biomarker genes**

Eight-hundred and fifty-one genes appear at least twice in sorafenib’s 48 linear ridge models. These genes constitute a starting set for identifying relationships between gene expressions and responses of either preclinical CGP IC_50_ or BATTLE clinical response. As with erlotinib, the aim is to identify subsets of gene expressions that separate sorafenib chemo-sensitive from chemo-resistant preclinical IC_50_, and also separate sorafenib treated BATTLE patients that have a clinically favorable versus unfavorable Months to Progression (i.e. responders versus non-responders). Students t-tests of the ~11k gene expressions common to the CGP sorafenib IC_50_ data and the sorafenib treated BATTLE patients finds 1313 genes with statistically significant expression differences (p<=0.05) when comparing the top and bottom 30^th^ percentiles of BATTLE clinical responders. A B-H correction finds that no genes satisfy the false discovery threshold of 0.05. Completing the same analysis of the sorafenib preclinical CGP IC_50_ data finds 564 of the 11,582 gene expressions satisfying p<=0.05, with none achieving a B-H threshold of 0.05. Only 124 of these genes (5.5%) are common to the statistically significant genes derived separately from the BATTLE clinical and CGP IC_50_ data. These results are consistent with the above findings for erlotinib; they do not strongly support a unified set of genes that can be jointly associated with sorafenib’s preclinical CGP IC_50_ values and with BATTLE clinical responses. Furthermore, only 104 of the 1313 significantly scoring genes using the BATTLE response and only 90 of the 564 significantly scoring genes using preclinical IC_50_ data are included in the 851 genes identified from the sorafenib linear ridge regression. (Intersection of the 104 and 90 are 11 genes). While relatively large numbers of statistically significant differential gene expressions are found using the Student’s t-test for sorafenib and erlotinib, relatively few of these genes intersect with those derived from linear ridge regression.

As found in the erlotinib analysis, clustered plots of the 104 and 90 genes expressions can, however, be used as indicators for roles of differential gene expressions in preclinical and clinical responses. For example, the left panel in **Fig 2** plots the clustered results for the pairwise Pearson correlation coefficients of the 104 genes derived jointly from the BATTLE dataset using Student’s t-tests and sorafenib linear ridge regression. The right panel displays the same information for the 90 genes derived jointly from the CGP IC_50_ data and linear ridge regression. The clustered plot in left panel finds a clear pattern of genes over expressed in patients with the best clinical response (lower left corner of the leftmost image) and genes overexpressed in patients with the worst clinical response (upper right corner of leftmost image). Summarizing the GSEA for the BATTLE-derived clustered plot finds only two GO: Molecular Function pathways; RECEPTOR ACTIVITY and LIGAND_DEPENDENT_NUCLEAR_RECEPTOR_ACTIVITY for the 33 genes relatively over expressed in BATTLE responders versus non-responders. The 71 genes over expressed in BATTLE non-responders versus responders are associated with DNA BINDING and KINASE BINDING pathways. The clustered plot in the right panel of **Fig 2** sorafenib segregates the 90 genes into two major clusters, each consisting of 45 genes. A majority of the genes in the lower left corner are over expressed in the chemo-sensitive CGP IC_50_ tumor cells. GSEA for these genes finds GO:Molecular Function Pathways comprised of RECEPTOR, TRANSFERASE and LIGASE ACTIVITY. No GSEA overlaps were found for the 45 over expressed genes associated with IC_50_ chemo-resistance. A broad interpretation of these findings suggests an association between sorafenib BATTLE responders and CGP IC_50_ chemo-response; with pathways associated with over expressed genes known to be targets of sorafenib. As observed with erlotinib, however, this type of analysis yields useful, yet not overly compelling, linkages between preclinical CGP IC_50_ data and BATTLE clinical responses.

**Fig 2.** Sorafenib: Left panel; clustered plot of pair-wise gene expression correlations for the 104 genes exhibiting statistical significance (p<=0.05) between the top and bottom 30^th^ percentile (responders versus non-responders, respectively) for sorafenib treated BATTLE patients and co-occurring in the 851 genes derived from linear ridge modeling. Right panel; clustered plot of pair-wise gene expression correlations for the 90 genes exhibiting statistical significance (p<=0.1) between the top and bottom 30^th^ percentile of chemo-response for sorafenib CGP IC_50_ and co-occurring in the 851 genes derived from linear ridge modeling. Color scheme represents the spectrum of correlation values (-1, blue to +1, red). Deeper colors in the left paned indicate a greater separation of correlation values between the BATTLE responders and non-responders, when compared to the CGP IC_50_ data.
