# Supplementary material for: A data mining approach for identifying pathway-gene biomarkers for predicting clinical outcome: A case study of erlotinib and sorafenib
Source: PLoS One. 2017 Aug 8;12(8):e0181991. doi: 10.1371/journal.pone.0181991 (PMC5549706; doi:10.1371/journal.pone.0181991)
Supplement: S3 Text — (DOC) [file pone.0181991.s009.doc]

**S3 Text**

**Erlotinib - Pathway fitness identified genes**

A detailed discussion of the genes associated with pathway meta-clusters for erlotinib is provided below. The first meta-cluster with positive pathway fitness scores (rows 15-23) consists of 23 genes. All of these genes are in the TRANSFERASE_ACTIVITY_TRANSFERRING_PHOSPHOROUS_CONTAINING_GROUPS pathway, with 40% or more of these genes appearing in the other 8 pathways in this meta-cluster. Included in this meta-cluster are 6 tyrosine kinases:

1. EGFR (Epidermal Growth Factor Receptor, or Receptor Tyrosine-Protein Kinase ErbB-1);a cell surface protein that binds to epidermal growth factor to induce receptor dimerization and tyrosine auto-phosphorylation leading to cell proliferation,
2. KDR (Protein-Tyrosine Kinase Receptor Flk-1); a type III receptor tyrosine kinase that functions as the main mediator of VEGF-induced endothelial proliferation, survival, migration and tubular morphogenesis,
3. LTK (Leukocyte Receptor Tyrosine Kinase); a member of the ROS/insulin receptor family of tyrosine kinases,
4. ALK (Anaplastic Lymphoma Receptor Tyrosine Kinase); a receptor tyrosine kinase which belongs to the insulin receptor superfamily,
5. ROR1 (Receptor Tyrosine Kinase-Like Orphan Receptor 1); a receptor tyrosine kinase-like orphan receptor that modulates neurite growth in the central nervous system and
6. TIE(Tyrosine Kinase With Immunoglobulin-Like And EGF-Like Domains); a tyrosine protein kinase that plays a critical role in angiogenesis and blood vessel stability.

Also included in this meta-cluster are two serine/threonine kinases (STK10 and STK11), an ephrin family member (EPHA5) and a polo-kinase (PLK4).

Analysis of the genes associated with the 9 pathways in meta-cluster(rows 15-23) finds STK11, STK10, MPP3, LTK, DGKE, HIPK3, MARK1 and CPNE3 as contributing the most to pathway fitness scores. Manuscript **Fig 5** summarizes these results. The bottom 4 genes in this list are relatively under expressed in the responder versus non-responder patients, while the top 4 genes are relatively over expressed in the responder versus non-responder patients. Literature supports roles for these genes in erlotinib efficacy. **STK11**(also known as LKB1)-deficient cells exhibit enhanced sensitivity to erlotinib *in vitro* and *in vivo*, an effect associated with alterations in energy metabolism and mitochondrial dysfunction, resulting in impaired ATP homeostasis and increased ROS . Relative **STK11** under expression in responders versus non-responders is consistent with this finding. **LTK** shares a high degree of homology (nearly 80% identical) with ALK and is thought to promote growth and survival through activation of RAS/MAPK and PI3K/AKT signaling pathways; an effect that would be expected to be diminished with relatively lower expression in responders compared to non-responders. **MPP3** is member of the family of membrane-associated proteins that interact with the cytoskeleton and regulate cell proliferation, signaling pathways, and intracellular junctions. PI3K can be activated by forming a complex with MAGuK-family proteins **MPP3**. Studies exploring the activation of PI3K/AKT/mTOR signaling in HPV-induced cancers find that erlotinib can induce growth delay of xenografted HPV-containing cervical carcinoma cells. Under expression of **MPP3** in BATTLE responders could contribute to reduced activation of PI3K and enhanced erlotinib efficacy.

Genes in meta-cluster(rows 15-23), that are relatively over expressed in responder versus non-responders include **HIPK3** (Fas-Interacting Serine/Threonine-Protein Kinase 3); a Homeodomain-Interacting Protein Kinase (HIPK) with roles in the transcriptional regulation, signal transduction, and regulation of protein stability, with a specific role in the transduction of proapoptotic signals by death receptors through interaction with TRADD and FADD. Relative over-expression may amplify **HIPK3**’s apoptotic role to produce a cellular condition that may enhance erlotinib sensitivity. **MARK1** (MAP/Microtubule Affinity-Regulating Kinase 1) is another relatively over expressed serine/threonine-protein kinase that is involved in cell polarity and microtubule dynamics regulation, with potential roles in cell cycle activation and DNA repair. Studies find **MARK1** overexpression in lung tumors . As a potential serine/threonine target of erlotinib, **MARK1** inhibition by erlotinib may disrupt these processes and enhance erlotinib efficacy. **DGKE** (Diacylglycerol Kinase Epsilon) is involved mainly in the regeneration of phosphatidylinositol (PI) from diacylglycerol in the PI-cycle during cell signal transduction. While a direct connection of **DGKE** over expression to erlotinib sensitivity could not be found in the literature, diacylglycerol-regulates protein kinase C (PKC), a family of serine/threonine kinases that has been shown to be involved in EGFR and KRAS signaling. **CPNE3** (Copine 3) is a calcium-dependent phospholipid-binding protein that mediates interactions between integrins and extracellular ligands, and plays a role in ERBB2-mediated tumor cell migration in response to growth factor heregulin stimulation . Notably, EGFR is not included within these top ranked genes, however three potential erlotinib serine/threonine/tyrosine kinase targets are included.

The top ranked genes contributing to pathway fitness scores for meta-cluster(rows 24-32) are **STK11**, **MARK1**, **DGKE**, described above, and **SMARCA5** and **RUVBL1**, the latter pair being relatively over expressed in responder versus non-responder patients. These two genes share helicase and ATPase activities; i) **SMARCA5** (SWI/SNF-Related Matrix-Associated Actin-Dependent Regulator Of Chromatin A5) is a member of the SWI/SNF family of proteins which have helicase and ATPase activities and are involved in cell proliferation and differentiation and ii) **RUVBL1** (TATA Binding Protein) possesses single-stranded DNA-stimulated ATPase and ATP-dependent DNA helicase (3 to 5) activity, where hexamerization is thought to be critical for ATP hydrolysis. Helicases are DNA-binding partners for EGFR-mediated transcriptional activation in the nucleus . Helicase over expression may contribute EGFR translocation to the nucleus and provide greater opportunities for erlotinib to inhibit transcriptional activation.

The top ranked genes contributing to pathway fitness scores for meta-cluster(rows 33-38) are ANAPC2, STK11, MARK1, MMP16, GCLM and WWP1, with the first two genes being relatively over expressed in the BATTLE non-responders versus responders. **ANAPC2**(Anaphase Promoting Complex Subunit 2), a member of the anaphase-promoting complex (APC), or cyclosome, is a ubiquitin ligase essential for mitotic progression. Over expression in BATTLE non-responders is consistent with a possible role of metaphase-anaphase transition in overcoming erlotinib activity, whereas under expression may mitigate mitotic progression and enhance erlotinib efficacy. Genes in this set that are relatively over expressed in responders compared to non-responders find novel linkages to erlotinib efficacy. **GCLM**(Glutamate-Cysteine Ligase Modifier Subunit), also known as gamma-glutamylcysteine synthetase, is the first rate limiting enzyme of glutathione synthesis. Because oncogenic alterations of metabolism render cancer cells addicted to nutrients, glutamine metabolism and its involvement in tumorigenesis may potentially be exploited for therapeutic purposes. For example, increased sensitivity of breast cancer cells treated with the polyamine analogue PG-11047 was associated with high expression levels of 9 biomarker proteins, inclusive of **GCLM**. While the role of **GCLM** in chemosensitivity is not known, **GCLM** has been implicated in the ER stress response, where targeting ER stress and AKT with OSU-03012 in combination with erlotinib or gefitinib has been found to overcome resistance to EGFR inhibitors. **WWP1** (TGIF-Interacting Ubiquitin Ligase 1), an E3 ubiquitin ligase, is a member of the family of WW domain-containing proteins that play important roles in the regulation of cellular functions such as protein degradation, transcription, and RNA splicing. The EGFR family consists of four members, including HER1/ErbB1, HER2/ErbB2, HER3/ErbB3, and HER4/ErbB4. **WWP1** selectively targets HER4 . Resistance to Cetuximab, an anti-EGFR monoclonal antibody, was overcome by targeting HER family receptors with antibody-based therapy . In addition, the irreversible pan-HER inhibitor HM781-36B has recently shown promise in the inhibition of EGFR, HER2, and HER4 . **MMP16** (Matrix Metallopeptidase 16) is a member of the matrix metalloproteinase (MMP) family which is involved in the breakdown of extracellular matrix in normal physiological processes, such as embryonic development, reproduction, and tissue remodeling . Elevated levels of matrix metalloproteinases (MMPs) were found in glioblastoma (GBM) cell-lines, as well as in GBM biopsies, and may well be a feature of some cancer cells, with no direct role in erlotinib efficacy. Targeting tumor-supportive cellular machineries is a relatively new part of anticancer drug development .

The meta-cluster(rows 1-14), with negative pathway fitness scores, consists of 30 genes where CACNG5, KCNJ3, NOX5 and CACNG1 (all relatively under expressed in responders versus non-responders) contribute the greatest to pathway fitness scores. **CACNG5** (Calcium Voltage-Gated Channel Auxiliary Subunit Gamma 5) is a type II transmembrane AMPA receptor regulatory protein (TARP). TARPs regulate both trafficking and channel gating of the AMPA receptors . An investigation of candidate gene biomarkers for sensitivity and resistance of human glioblastoma multiforme cell lines to erlotinib found **CACNG4** to be included in the 10 biomarker genes associated with resistance to erlotinib. **CACNG4** under expression may mitigate this resistance and enhance erlotinib sensitivity. **KCNJ3** (Potassium Voltage-Gated Channel Subfamily J Member 3) is a member of the family of potassium channels. Voltage-gated K(+) (Kv) channels are associated with the proliferation of several types of cancer cells, including lung adenocarcinoma cells, with some Kv channel blockers inhibiting cancer cell proliferation. Relative under expression of **KCNJ3** in erlotinib responders may compromise tumor cell proliferation in a fashion equivalent to Kv blockers. **NOX5** (NADPH Oxidase, EF-Hand Calcium Binding Domain 5) encodes a calcium-dependent NADPH oxidase that generates superoxide, and functions as a calcium-dependent proton channel that may regulate redox-dependent processes . Elevated NADPH oxidase levels have been found in lung cancer tumors and proposed to contribute to high basal ROS in cancer and poor survival . Over expression of **NOX5** in erlotinib non-responders is consistent with this premise, whereas under expression in responders may contribute to erlotinib sensitivity.

Manuscript **Fig 6** displays the pathway fitness results for the genes selected in meta-cluster(rows 49-55), with negative fitness scores. This meta-cluster has two genes as top ranked contributors to pathway fitness (DUSP6 and SBF1), both relatively over expressed in non-responders versus responders. **DUSP2** (Dual Specificity Phosphatase 2) is a member of the dual specificity protein phosphatase subfamily that inactivates their target kinases by dephosphorylating both the phosphoserine/threonine and phosphotyrosine residues. They negatively regulate members of the mitogen-activated protein (MAP) kinase superfamily (MAPK/ERK, SAPK/JNK, p38), which are associated with cellular proliferation and differentiation. Their relative under expression in BATTLE responders versus non-responders is consistent with the roles of dual specificity phosphatases in tumor responses to drugs that target Ras/ERK. **SBF1** (SET Binding Factor 1) is a member of the protein-tyrosine phosphatase family. However, the encoded protein does not appear to be a catalytically active phosphatase because it lacks several amino acids in the catalytic pocket. This protein contains a Guanine nucleotide Exchange Factor (GEF) domain which is necessary for growth and differentiation . Over expression of GEFs in erlotinib-resistant cell lines increased NFκB activation in several different types of cancer cells . Manuscript **Table 1** summarizes selected pathway-fitness-selected genes for the erlotinib meta-clusters.
