# Supplementary material for: A data mining approach for identifying pathway-gene biomarkers for predicting clinical outcome: A case study of erlotinib and sorafenib
Source: PLoS One. 2017 Aug 8;12(8):e0181991. doi: 10.1371/journal.pone.0181991 (PMC5549706; doi:10.1371/journal.pone.0181991)
Supplement: S4 Text — (DOC) [file pone.0181991.s010.doc]

**S4 Text**

**Sorafenib - Pathway fitness identified genes**

A detailed discussion of the genes in pathway meta-clusters for sorafenib is presented below. Meta-cluster(rows 13-18), with positive H scores, contain pathway genes also found in meta-cluster(rows 1-5), inclusive of NEK11, TRIB1 and SPHK1. Additional genes identified as having lesser, yet top-ranked, contributions to pathway scores include **RRAGB** and **BMPR1B**, both showing over expression in BATTLE responders when compared to non-responders. **RRAGB** (Ras-Related GTP Binding B) is part of the GTPase family of signal transducers that alternate between an activated, GTP-binding state and an inactivated, GDP-binding state. **BMPR1B** (Bone Morphogenetic Protein Receptor Type 1B), which encodes a member of the bone morphogenetic protein (BMP) receptor family of transmembrane serine/threonine kinases. While serine/threonine protein kinases are targeted by sorafenib, they function downstream of the Ras subfamily of membrane associated GTPases. These results suggest that sorafenib may either have an effect on the Ras pathway, or over expression of Ras family members may contribute to sorafenib efficacy.

Meta-cluster(rows 40-43), with positive H scores, involve pathways containing primarily chemokine ligands (CCL8, CCL20, CCL22, CCL18, CXCL9 and CXCL10), with the top-ranked contribution to pathway fitness scores from only **CCL20**, with relative over expression in BATTLE responders compared to non-responders. A role for cytokine therapy enhancing sorafenib efficacy has been noted in the treatment of renal cell cancer . Sorafenib also sensitizes HCC (Human Hepatocellular Carcinoma Cells) to the apoptotic activity of Transforming Growth Factor-β(TGF-β) through the intrinsic pathway and to Tumor Necrosis Factor-α (TNF) through the extrinsic pathway. Sorafenib effectiveness in delaying HCC progression might be partly related to a selective sensitization of HCC cells to apoptosis by disrupting autocrine signals that protect them from adverse conditions and pro-apoptotic physiological cytokines . Over expression of the cytokine **CCL20** may contribute to the enhanced sorafenib effect.

Meta-cluster(rows 6-10),with negative H scores, involve TRANSPORTER pathways, mainly comprised of the family of solute carriers (SLC5A6, SLC5A1, SLC13A4, SLC12A1, SLC34A1 and SLC16A7). Top ranked genes contributing to pathway scores include **SLC5A1** and **SLC1A4**, which are over expressed in the responder versus non-responder patients. Over 400 SLC transporter genes have now been identified, representing 55 families, including ion coupled transporters, exchangers and passive transporters located at the plasma membrane or in intracellular organelles. These super families are responsible for mediating the transport of a wide spectrum of substrates, including nutrients and drugs. Cancer cells with enhanced expression of SLC transporters for certain nutritional requirements may provide a growth advantage over normal cells when nutrients become restricted. Sorafenib does not appear to rely on active transport to enter the cell, nor is it a substrate for ABC efflux transporters. Consequently the role of SLC over-expression in sorafenib BATTLE responders does not appear to be related to transporter-mediated alterations of drug influx . A more likely possibility is due to the recent finding that multi-kinase inhibitors also selectively inhibit solute carriers .

Top ranked genes contributing to negative pathway scores for meta-cluster(rows 6-10), that are over expressed in non-responders versus responders, include **SEC61B**; necessary for protein translocation in the endoplasmic reticulum, **COX4I1** (Cytochrome C Oxidase Subunit IV) and **COX7A1**; terminal oxidases in mitochondrial electron transport, **KCNK3**(Potassium Channel, Two Pore Domain Subfamily K) and **KCNC3**(Potassium Voltage-Gated Channel Subfamily C Member 3); members of the superfamily of potassium channel proteins. Support for roles of these genes in sorafenib resistance exists in the literature. Mitochondrial metabolism greatly influences cancer cell survival, invasion, metastasis, and resistance to many anticancer drugs such that molecular-targeted therapies (e.g., oncogenic kinase inhibitors) create a dependence of surviving cells on mitochondrial metabolism. Inhibition of mitochondrial metabolism represents a promising therapeutic target in cancer, inclusive of members of the mitochondrial electron transport chain. In addition, evasion of drug-induced apoptosis has been demonstrated with an upregulation of ion transporters . Over expression of **KCNK3** and **KCNC3** is associated with BATTLE non-responders (i.e. sorafenib resistance).

Meta-cluster(rows 35-39), with negative H scores, involve pathways that modulate kinase activity by inhibition, binding or regulation. Genes in this meta-cluster include Forkhead Box O3 (**FOXO3**) and the family of cyclin-dependent kinase inhibitors (**CDKN2D**, **CDKN2C**), as the greatest contributors to pathway fitness scores. Support for the over expression of these genes in sorafenib resistance can be found in the literature. In K562 chronic myelogenous leukemia cells the acquisition of chemo-resistance correlated with the increased expression and nuclear accumulation of **FOXO3a**, via a mechanism involving enhanced PI3K/AKT activity. Moreover, the induction of **FOXO3a** activity in naïve K562 cells was sufficient to enhance PI3K/AKT activity and to confer resistance to the cytotoxic effects of doxorubicin. A role for CDKs in drug sensitivity was recently reported from studies into a role for nuclear protein 1 (NUPR1) over expression in primary human HCC samples versus normal liver where knockdown of NUPR1 significantly increased cell sensitivity to sorafenib and inhibited the cell growth, migration and invasion of HCC cells, both *in vitro* and *in vivo*. Gene expression profiling of HCC cells following stable NUPR1 knockdown, found suppression of genes functionally involved in cell death and survival, cellular response to therapies, lipid metabolism, cell growth and proliferation, molecular transport and cellular movement. Included in these suppressed genes was **CDKN2**, suggesting a reversal of sorafenib sensitivity in the presence of elevated CDKs. This result is consistent with relative CDK overexpression in non-responders versus responders. Manuscript **Table 2** summarizes selected pathway-fitness-selected genes for the sorafenib meta-clusters.
