# Supplementary material for: A data mining approach for identifying pathway-gene biomarkers for predicting clinical outcome: A case study of erlotinib and sorafenib
Source: PLoS One. 2017 Aug 8;12(8):e0181991. doi: 10.1371/journal.pone.0181991 (PMC5549706; doi:10.1371/journal.pone.0181991)
Supplement: S5 Text — (DOC) [file pone.0181991.s011.doc]

**S5 Text**

**Exploring pathway fitness scores across different gene sets**

To address this issue, pathway fitness scores, based on GSEA derived genes (n=251), were compared to fitness scores obtained using 4627 of the ~11k erlotinib genes that exist in the complete set of 396 GO:Molecular Function pathways. **Fig 3** displays the fitness scores as they appear in manuscript **Fig 4** with fitness scores determined from the expanded gene set. Evident from this plot is a good correlation (r=0.843, p=1.652e-17) between these sets of fitness scores. This result supports the calculation of fitness scores using the reduced set of genes.


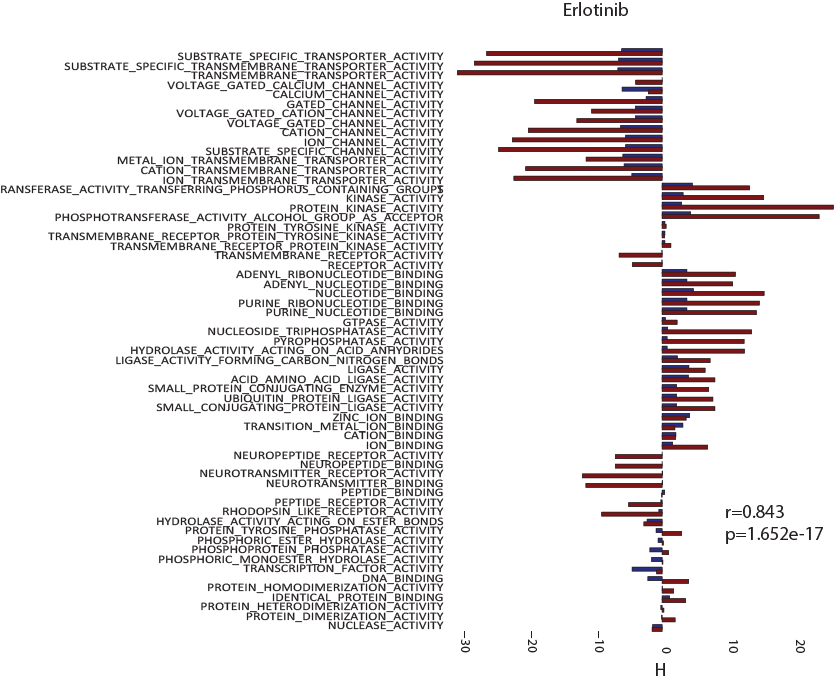


**Fig 3.** Comparison of fitness scores calculated using the 251 genes GSEA-derived genes (blue) and using the 4627 genes that exist in the 396 GO:Molecular Function pathways (red). Correspondence is best for the genes in the uppermost pathways. Conclusion: fitness scores are robust across gene sets. Pearson correlation statistics r=0.843, p=1.652e-17.

Pathway fitness scores based on GSEA derived genes (n=309) can be compared to scores obtained using 4820 of the ~11k sorafenib genes that exist in the complete set of 396 GO:Molecular Function pathways. **Fig 4** displays the fitness scores as they appear in manuscript **Fig 5** with fitness scores determined from the expanded gene set. Evident from this plot is a good correlation (r=0.652, p=1.196e-6) between these sets of fitness scores. This result supports the calculation of fitness scores using the reduced set of genes.

**
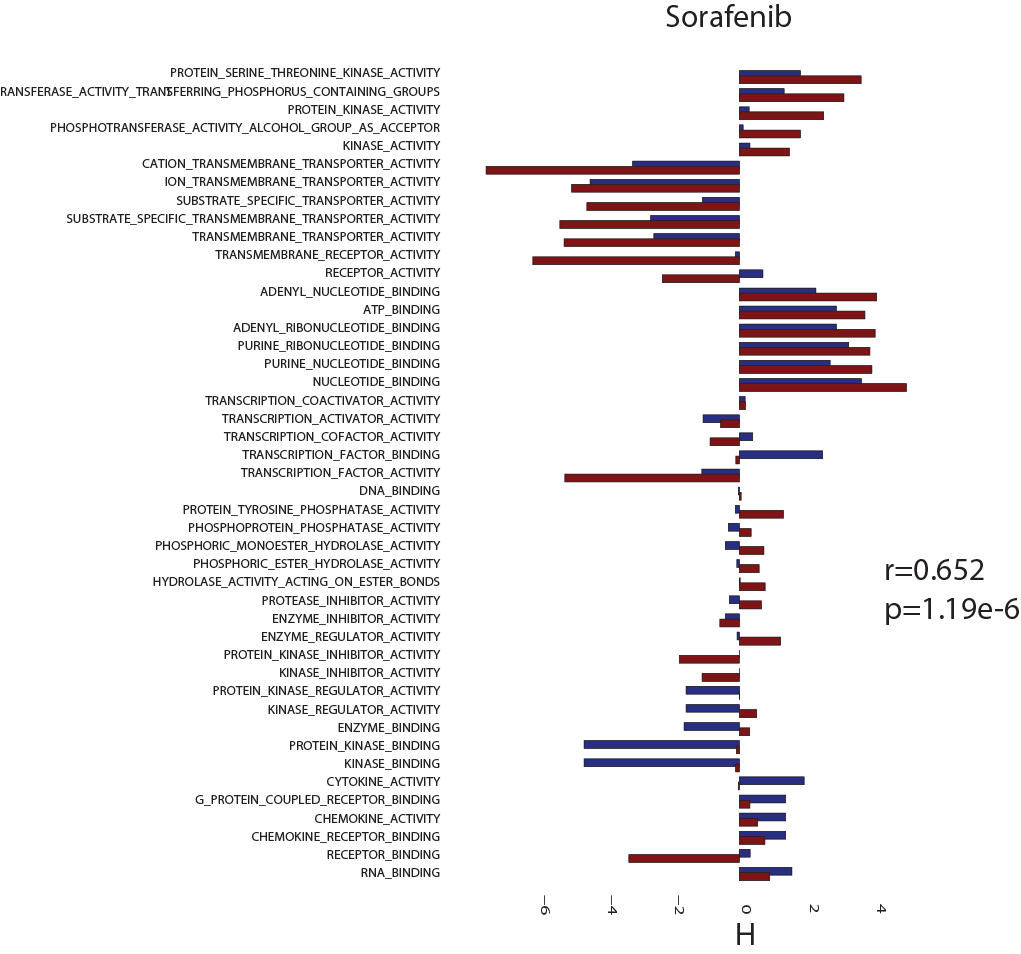
**

**Fig 4.** Comparison of fitness scores calculated using the 309 genes GSEA-derived genes (blue) and using the 4580 genes that exist in the 396 GSEA GO:Molecular Function pathways (red). Correspondence is best for the genes in the uppermost pathways. Conclusion: fitness scores are robust across gene sets. Pearson correlation statistics r=0.652, p=1.19e-6.
